# Ultra-fast label-free quantification and comprehensive proteome coverage with narrow-window data-independent acquisition

**DOI:** 10.1038/s41587-023-02099-7

**Published:** 2024-02-01

**Authors:** Ulises H. Guzman, Ana Martinez-Val, Zilu Ye, Eugen Damoc, Tabiwang N. Arrey, Anna Pashkova, Santosh Renuse, Eduard Denisov, Johannes Petzoldt, Amelia C. Peterson, Florian Harking, Ole Østergaard, Rasmus Rydbirk, Susana Aznar, Hamish Stewart, Yue Xuan, Daniel Hermanson, Stevan Horning, Christian Hock, Alexander Makarov, Vlad Zabrouskov, Jesper V. Olsen

**Affiliations:** 1 Novo Nordisk Foundation Center for Protein Research, University of Copenhagen, Copenhagen, Denmark; 2 State Key Laboratory of Common Mechanism Research for Major Diseases, Suzhou Institute of Systems Medicine, Chinese Academy of Medical Sciences & Peking Union Medical College, Suzhou, China; 3 Thermo Fisher Scientific (Bremen) GmbH, Bremen, Germany; 4 Thermo Fisher Scientific, San Jose, CA USA; 5 Center for Functional Genomics and Tissue Plasticity (ATLAS), Department of Biochemistry and Molecular Biology, University of Southern Denmark, Odense, Denmark; 6 Centre for Neuroscience and Stereology, Copenhagen University Hospital, Copenhagen, Denmark

**Keywords:** Proteomic analysis, Mass spectrometry

## Abstract

Mass spectrometry (MS)-based proteomics aims to characterize comprehensive proteomes in a fast and reproducible manner. Here we present the narrow-window data-independent acquisition (nDIA) strategy consisting of high-resolution MS1 scans with parallel tandem MS (MS/MS) scans of ~200 Hz using 2-Th isolation windows, dissolving the differences between data-dependent and -independent methods. This is achieved by pairing a quadrupole Orbitrap mass spectrometer with the asymmetric track lossless (Astral) analyzer which provides >200-Hz MS/MS scanning speed, high resolving power and sensitivity, and low-ppm mass accuracy. The nDIA strategy enables profiling of >100 full yeast proteomes per day, or 48 human proteomes per day at the depth of ~10,000 human protein groups in half-an-hour or ~7,000 proteins in 5 min, representing 3× higher coverage compared with current state-of-the-art MS. Multi-shot acquisition of offline fractionated samples provides comprehensive coverage of human proteomes in ~3 h. High quantitative precision and accuracy are demonstrated in a three-species proteome mixture, quantifying 14,000+ protein groups in a single half-an-hour run.

## Abstract (web summary)

A new mass spectrometry strategy combines the advantages of data-dependent and data-independent acquisition.

## Main

Genomics and proteomics offer immense potential for enhancing human health and the environment. For example, next-generation sequencing has revolutionized genomics by making DNA and RNA sequencing much faster and cost-effective. Conversely, MS-based proteomics has only gradually improved MS hardware, acquisition strategies and software^1,2^, to enable comprehensive profiling of human cell line proteomes, encompassing nearly all expressed proteins^3,4^. However, scalability remains a challenge, as in-depth proteome coverage requires extended MS measurement time^4,5^, limiting its application in systems biology and large clinical cohort studies, where coverage of low-abundance transcription factors and signaling proteins is often sacrificed for throughput. MS data acquisition has shifted from data-dependent acquisition (DDA) to data-independent acquisition (DIA)^1,6,7^. Faster and more sensitive MS instruments, along with advanced data processing software, have made DIA the preferred method for maximizing proteome coverage in label-free single-shot analysis^8,9^. For instance, the Orbitrap analyzer’s acquisition speed has increased notably, from 3–5 Hz for the original LTQ Orbitrap mass spectrometer^2^ to 50 Hz, while time-of-flight (ToF) mass spectrometers routinely generate more than 100 averaged MS/MS scans per second^10^.

Here, we introduce narrow-window data-independent acquisition (nDIA) which combines high-resolution MS1 scans with parallel, ultra-fast MS/MS scans of ~200 Hz with high resolution and sensitivity. This approach empowers the use of narrow 2-Th DDA-like isolation windows for nDIA, enabling comprehensive peptide precursor coverage. We demonstrate that single-shot analysis facilitates comprehensive proteome profiling and is ideal for high-throughput proteomics. For human proteome profiling, we introduce an optimized method that swiftly generates comprehensive proteomes by combining offline high-pH (HpH) reversed-phase peptide fractionation and short online liquid chromatography (LC) gradients (180 samples per day (SPD)) with nDIA. This method achieves nearly complete coverage of the expressed human proteome of ~12,000 proteins within 3–4.5 h of analysis.

## Results

### Orbitrap Astral MS blurs the contrast between DIA and DDA

Clinical proteomics and systems biology studies require high-throughput liquid chromatography with tandem MS (LC–MS/MS) analyses with deep proteome coverage and accurate quantification. To achieve this, it is necessary to reduce MS measurement time by deploying shorter LC gradients with faster scanning MS instruments that can cope with the higher sample complexity per unit time. DIA has become the method of choice for single-shot deep proteome profiling with short gradients due to its high reproducibility and coverage and excellent quantitative performance^7,11^.

**Fig. 1:**
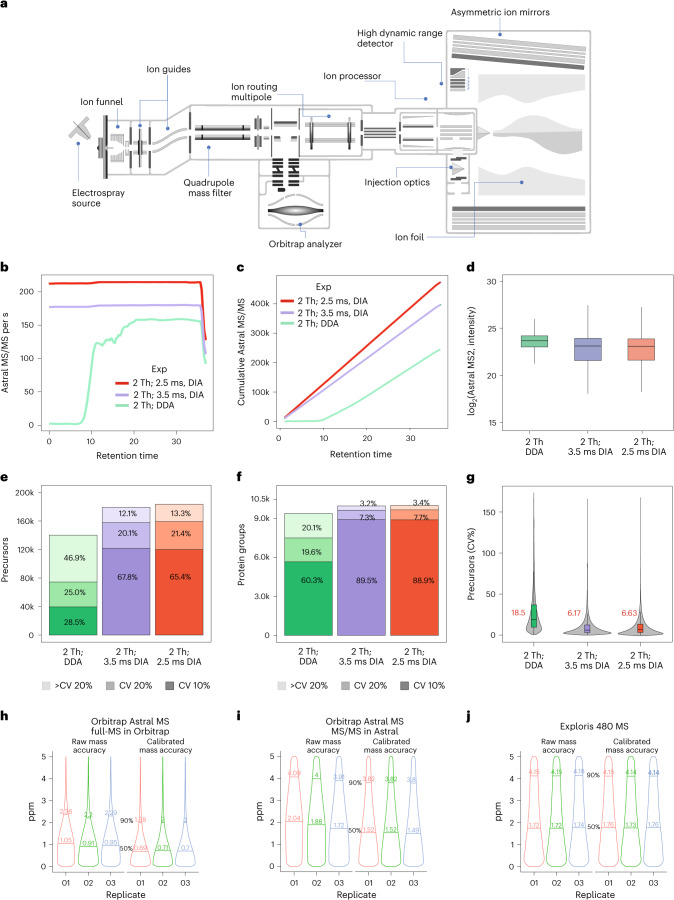
Benchmarking of nDIA against DDA. **a**, Hardware overview of the Orbitrap Astral MS instrument. **b**, MS/MS scan rate over the 28-min effective chromatographic gradient length. DDA and DIA slow (3.5 ms) and fast (2.5 ms) methods are depicted by different colors (green, purple and red, respectively). **c**, Cumulative MS/MS scans across the 28-min effective chromatographic gradient acquired in the Astral analyzer. **d**, log_2_ intensity of MS/MS spectra measured in the Astral analyzer with DDA and DIA. **e**, Quantified precursors measured with DDA and nDIA operation mode below 10% and 20% of CV (*n* = 3, 1,000 ng of tryptic HEK293 peptides). **f**, Quantified and identified proteins below 10% and 20% of CV with DDA and nDIA approaches (*n* = 3, 1,000 ng of tryptic HEK293 peptides). **g**, CVs (%) for quantified precursors by different operation modes. Median CV is shown in red for each approach. **h**–**j**, Absolute median mass deviation of peptide precursors determined with the Orbitrap Astral full-MS using the Orbitrap analyzer (**h**), the Orbitrap Astral MS/MS using the Astral analyzer (**i**) and the Orbitrap Exploris 480 MS using the Orbitrap analyzer (**j**); 50th and 90th quantiles are shown. In boxplot figures, center lines show the medians; box limits indicate the 25th and 75th percentiles; whiskers extend 1.5 times the interquartile range from the 25th and 75th percentiles. Source data

In DIA, co-eluting peptide ions are co-isolated and fragmented in predefined mass isolation windows, resulting in complex spectra containing fragment ions from multiple peptides. Conversely, DDA has higher specificity due to narrower isolation windows but with inferior peptide sequencing capacity. Although DIA can achieve higher specificity by constraining the quadrupole isolation width similar to DDA, state-of-the-art mass spectrometers cannot provide the sensitivity and acquisition speed needed to routinely perform DIA with 2-Th isolation windows on a chromatographic time-scale. An improved mass analyzer is required to address the trade-off between speed versus sensitivity in the Orbitrap and limited ion transmission in conventional ToF analyzers. Here, we leverage the asymmetric track lossless (Astral) mass analyzer, capable of ~200-Hz MS/MS acquisition rates at high resolving power and sensitivity, which permits routine nDIA analysis with narrow 2-Th isolation windows. The analyzer is a part of the Thermo Scientific Orbitrap Astral mass spectrometer^12^, which combines a modified Thermo Scientific Orbitrap Exploris 480 (ref. ^13^) mass spectrometer with the Astral analyzer^12,14^^,15^ via a transport octapole appended on the end of the Orbitrap Exploris ion routing multipole (IRM) (Fig. 1a, Supplementary Fig. 1 and Supplementary Note 1).

In MS/MS acquisition, ions are first accumulated in the IRM, and then directed across to the ion processor, a linear quadrupole ion trap incorporating two pressure regions. The function of the ion processor is similar to the combination of a C-Trap and IRM, albeit operating much faster. Admitted ions are accumulated and fragmented via higher-energy collisional dissociation (HCD)^16^ in a high-pressure region, and then passed at low energy to a low-pressure region, across an apertureless interface, before final cooling and pulsed extraction to the Astral analyzer. The two regions operate in a parallelized manner such that two ion packets are processed simultaneously, in addition to a third within the IRM and any being detected with the Orbitrap and Astral analyzers, for a total of five ion packets. This approach enables the alignment of a high spectral acquisition rate with a relatively long ion processing time in the low-millisecond range. The performance envelope is particularly well suited to MS/MS operation, and in a typical analysis the Astral analyzer is set to handle all MS/MS acquisition. The coupled Orbitrap analyzer, on the other hand, is given the full length of the MS/MS cycle to acquire parallel full-MS spectra, which allows it to run at 240,000 or 480,000 resolution settings much higher than the typical 60,000 or 120,000, and which also improves full-MS sensitivity and resolution.

It is possible to operate the instrument in DDA mode with top speed acquisition of ~150 Hz, but restricting maximum ion fill time to 3.5 ms in nDIA mode results in 170-Hz MS/MS acquisition rate while injection time of maximum 2.5 ms delivers >200-Hz MS/MS acquisition (Fig. 1b and Supplementary Fig. 2a,b). The fastest nDIA scanning speed generates ~400,000 DIA MS/MS spectra in half-an-hour of LC–MS/MS analysis of a human embryonic kidney cell (HEK293) tryptic digest (Fig. 1c). The overall MS/MS spectra intensity was higher in DDA mode (Fig. 1d), reflecting the abundance bias of DDA methods having the propensity to isolate the most abundant precursors^17^ (Supplementary Fig. 2c). Despite lower MS/MS intensities, nDIA acquisition mode resulted in higher identifications of approximately 170,000 peptide precursors and ~10,000 protein groups (hereafter referred to as proteins) (Fig. 1e,f). The quantitation of peptides and protein identifications can be performed with high reproducibility, with coefficient of variation (CV) below 20% for 90% of precursors and 95% of proteins, respectively (Fig. 1e,f). The median CVs at precursor level for nDIA were lower compared with DDA (<7% versus <19%, respectively) (Fig. 1g). This observation can likely be explained by the semi-stochastic nature of DDA precursor selection^18^. The comparison between DDA and DIA strategies showed higher performance for nDIA, although closely followed by DDA and also with faster LC gradients and different DDA acquisition schemes with 15-min LC–MS/MS runs (Supplementary Fig. 2d–f). Of the four DDA acquisition methods compared, three of them, top 100, top 75 and cycle 0.5 s, showed comparable performance, with >70,000 quantified precursors in 15 min at the top speed acquisition of ~150 Hz. However, DDA cycle 0.5 s and top 75 showed the best protein identifications, with 7,378 and 7,061 quantified proteins, respectively. Despite that the DIA-NN software was originally designed for DIA data, it is superior to most DDA-specific search engines for analyzing Orbitrap Astral DDA data. We therefore used DIA-NN for analysis of both DIA and DDA data to maintain the same bioinformatics workflow for both methods.

To ensure reliable identifications from the large number of DIA MS/MS scans recorded with the Astral analyzer, we estimated the empirical false discovery rate (FDR) using entrapment database analyses^19^ with spectral library-free searches in Spectronaut (v.18). First, we searched against a 9× larger shuffled human mimic database. The empirical FDR at protein level corrected for the mimic database size was estimated to be ~0.7% when analyzing both HEK293 and HeLa datasets (Supplementary Fig. 3a,b). Second, we also estimated empirical FDR by searching against an *Arabidopsis thaliana* database as decoy and performed FDR estimations by conducting bootstrap analyses with 23 repetitions. Reassuringly, the corrected FDR estimation from this was 1.25%, consistent with the mimic database entrapment analysis (Supplementary Fig. 3c,d). Furthermore, by using Spectronaut’s algorithm for interference detection and analyzing all precursors identified, we found that only 1.4% had interfering ions (Supplementary Fig. 3e,f).

Mass accuracy determines fragment ion identity in peptide search engines^20^. To evaluate the Astral mass analyzer’s accuracy in MS/MS mode, we used a 200-Hz, 2-Th nDIA analysis of a tryptic digest of HEK293 cell lysate. The Orbitrap MS1 full scan at 240,000 resolution showed 0.95-ppm mass median absolute deviation (Fig. 1h), akin to previous reports^21^. For matched fragment ions recorded by the Astral analyzer, the median absolute deviation was 1.88 ppm, similar to Orbitrap MS/MS at 15,000 resolution (Fig. 1i,j). Although the Orbitrap mass calibration remains stable for days, the Astral mass accuracy may drift (~3 ppm) over time. This drift can be corrected through regular automatic recalibration via the internal calibrant source or post-acquisition recalibration, resulting in sub-ppm-level mass accuracy.

### Fast sequencing enables deep proteome profiling

Comprehensive proteome coverage is essential for large-scale systems biology studies involving thousands of conditions. The first complete proteome of a eukaryotic organism analyzed by MS was that of yeast^22^, and with state-of-the-art instrumentation it was possible to cover ~4,000 yeast proteins in approximately 1 h of LC–MS/MS time^23^.

**Fig. 2:**
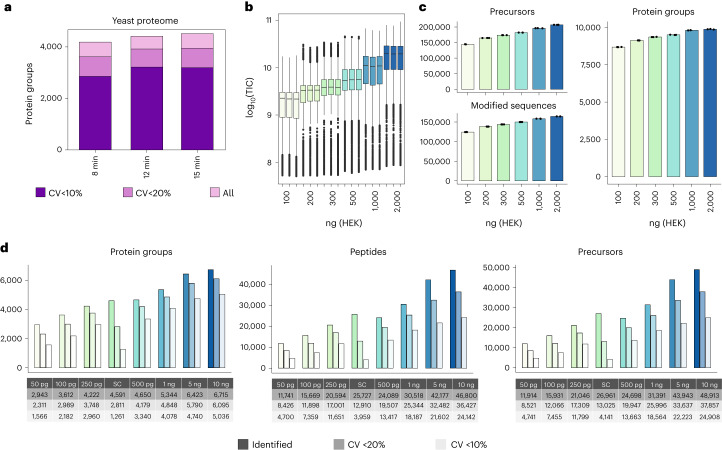
Deep single-shot proteome coverage from high to low sample loads. **a**, Protein groups identified and quantified below 10% and 20% of CV (*n* = 3) in a yeast extract using different chromatographic gradient lengths. **b**, Boxplot of total ion current (in log_10_ scale) measured in a dilution series (from 100 to 2,000 ng) of a HEK293 cell protein digest. **c**, Protein groups, precursors and modified peptides identified (*n* = 3) in a dilution series of HEK293 cell peptide extract using Spectronaut (v.17). **d**, Protein groups, peptides and precursors identified in three replicates in a low-amount dilution series and 12 single HeLa cells (SC) individually using Spectronaut (v.18). Next to each bar, the number of each quantified with CV < 20% and CV < 10%. Box limits indicate the 25th and 75th percentiles as determined by R software; whiskers extend 1.5 times the interquartile range from the 25th and 75th percentiles; outliers are represented by dots. TIC, total ion chromatogram. Source data

Analyzing whole yeast cell digests with nDIA (2-Th) across various LC gradient lengths, from 5-min active LC gradients (180 SPD) up to 96 SPD (15-min gradient), achieved nearly complete yeast proteome coverage (~4,500 proteins) ten times faster than previous reports, and maintaining high quantitative reproducibility (Fig. 2a).

**Extended Data Fig. 1:**
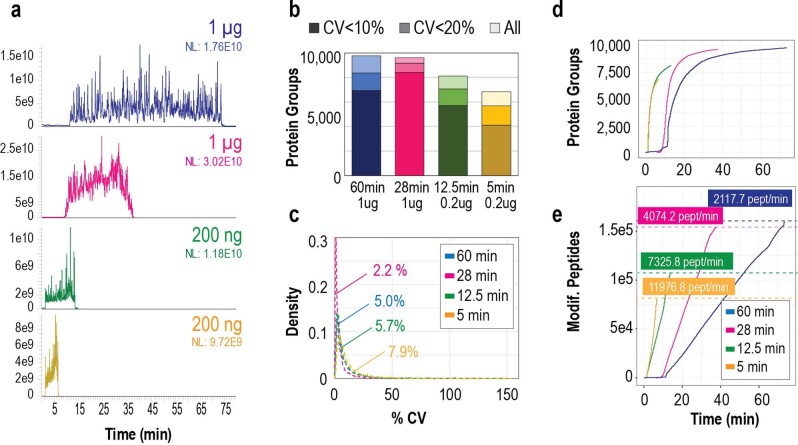
Optimization of DIA acquisition parameters. (**a**) Total ion chromatograms of different MS runs of HEK293 peptide extracts. From top to bottom: 60 minutes and 1 µg load, 28 minutes and 1 µg load, 12.5 minutes and 200 ng load and 5 minutes and 200 ng load. (**b**) Protein Groups identified and quantified below 10 and 20% of coefficient of variation (n = 3) in different loadings and gradients of HEK293 cells peptide extract using Spectronaut (v18). (**c**) Distribution of protein groups quantitation coefficient of variation for each gradient. Number in the plot indicates the median CV. (**d**, **e**) Cumulative identifications of protein groups (d) or modified peptides (e) for 1 µg in 28 or 60 minutes of chromatographic separation, or 200 ng in 12.5 or 5 minutes of chromatographic separation. Source data

In contrast, a human cell proteome comprises >12,000 (refs. ^3,4,24^) protein-coding genes with a high dynamic range (>6-logs)^25,26^, demanding extended MS measurement time for comprehensive coverage. To evaluate human proteome coverage and sensitivity achieved with nDIA (2-Th), we measured increasing amounts of HEK293 digests (from 100 ng to 2,000 ng) with 60-min active LC gradients. Reassuringly, the measured MS/MS signal scaled with loading amounts and did not seem to be saturated at the highest load (Fig. 2b). Peptide and protein coverage and quantitative reproducibility also correlated with loading amounts, with an average of 9,879 proteins and 159,627 unique peptides (164,216 modified peptides) when injecting 2 µg of HEK293 protein digest, but covering 88% of proteins with only 100 ng (Fig. 2c and Supplementary Table 1). To optimize proteome coverage and MS measurement time, we compared different loadings of the HEK293 digest across different LC gradient lengths (Supplementary Table 2, Extended Data Fig. 1 and Supplementary Note 2). This analysis revealed that the 28-min active LC gradient of 1 µg resulted in 9,619 proteins with outstanding reproducibility (median CV < 3%) (Supplementary Table 3).

Reduced identifications with lower sample loads highlight the necessity for tailored DIA acquisition methods, especially for high-sensitivity strategies. To address this, we widened the isolation window and scaled the maximum injection time (maxIT) for decreasing sample amounts, while maintaining a constant scan cycle time (Supplementary Fig. 4a). We analyzed 10-ng, 25-ng and 100-ng injections using 2-Th, 4-Th, 8-Th and 16-Th windows, with 3.5-ms, 7-ms, 14-ms and 28-ms maxITs (Supplementary Table 2), respectively, using a 60-min active LC gradient. We found that for 100 ng, the optimal method was 4-Th windows with 7-ms maxIT, yielding 8,600 proteins. Lower loads benefited from wider 8-Th windows, resulting in 6,600 proteins from just 10-ng input (Supplementary Fig. 4b and Supplementary Table 4). Decreasing loading amounts rarely reached the ideal ion target value and this under-sampling is most pronounced with narrow isolation windows (Supplementary Fig. 4c). The impact of different window sizes was not uniform across the mass range, with peptides of higher *m*/*z* most affected (Supplementary Fig. 4d). Therefore, scaling window size and maxIT in different mass ranges according to sample loads may provide a means for maximizing peptide coverage given the sample input. To test the instrument sensitivity, we performed DIA analyses on a range of HeLa digest dilutions, spanning from 50 pg up to 10 ng, and also included analysis of 12 individual single HeLa cell preparations (Fig. 2d). To maximize sensitivity of the MS/MS scans, the DIA isolation windows were gradually widened from 5 Th to 20 Th, scaling maxIT accordingly, and using high-Field Asymmetric waveform Ion Mobility Spectrometry (FAIMS) with single compensation voltage of −50 V optimized for DIA-based proteome analyses ^13^. The reason for adopting this approach was to improve coverage of peptides with lower abundance, otherwise missed with narrower isolation windows, while FAIMS acted as an ion filter to minimize singly charged background ions, thereby enhancing multiply charged peptide signal-to-noise ratios. As expected, peptide and protein coverage scaled with input amount, but reproducible quantification was maintained even in the low-abundance range (Fig. 2d). These data establish the high sensitivity of the Orbitrap Astral MS instrument, enabling the identification of ~3,000 proteins from 50 pg and >4,500 proteins from single HeLa cells. Approximately 80% of these proteins were quantified with CV < 20% from 250 pg in dilution series. The single-cell data exhibited higher variation, which is expected due to the inherent heterogeneity of individual cells. This dataset demonstrates that the Orbitrap Astral MS instrument is particularly well suited for single-cell proteomics research.

To benchmark the performance of the nDIA strategy on the Orbitrap Astral for high-throughput proteomics against the current state-of-the-art, we compared the 5-min HeLa analysis results with reference DIA datasets on HeLa recorded with an Orbitrap Exploris 480 (ref. ^13^), a ZenoTOF 7600 (ref. ^27^), a TripleTOF 6600 (ref. ^8^) or a timsTOF HT (ref. ^28^), also with 5-min LC gradients, respectively. When analyzed with the same DIA processing software tools (Spectronaut and DIA-NN) and software settings rendering the results comparable, the Orbitrap Astral MS instrument identified 7,538 protein groups using DIA-NN in spectral library-free mode, whereas the TripleTOF identified 3,330 proteins, the ZenoTOF 3,419 proteins, the timsTOF HT 3,737 proteins and the Orbitrap Exploris identified 3,143 proteins (Supplementary Fig. 5a). The nDIA analysis with the Orbitrap Astral reproducibly identified >75,000 different modified peptide variants with the 5-min gradient, which was 3× more identifications compared with any of the other MS platforms (Supplementary Fig. 5b).

### nDIA enables precise and accurate label-free quantification

**Fig. 3:**
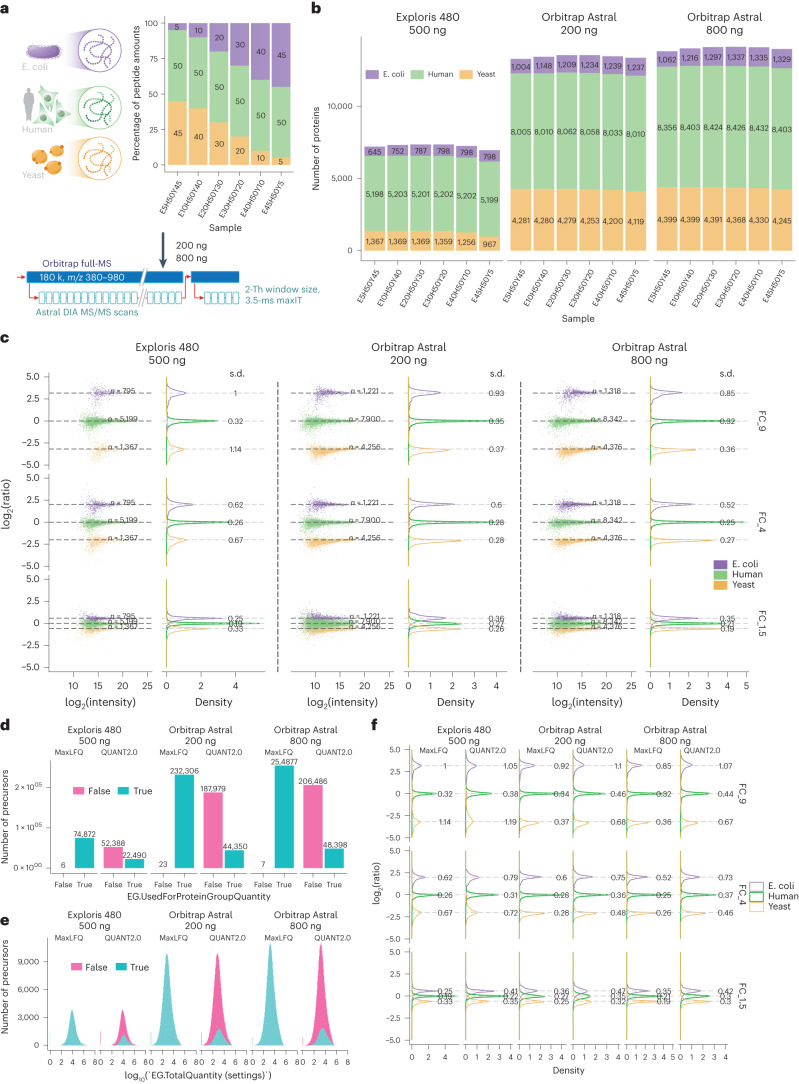
Enhanced LFQ accuracy and precision with the Orbitrap Astral mass spectrometer. **a**, Graphical representation of the experimental design. Tryptic peptides from three species were combined in six distinct ratios (E5H50Y45, E10H50Y40, E20H50Y30, E30H50Y20, E40H50Y10 and E45H50Y5). Samples were processed using the Orbitrap Astral mass spectrometer in technical triplicates, employing a 3.5-ms maxIT and 2-Th window size method. The loading amounts were 200 ng and 800 ng. **b**, Number of proteins identified from the three species in each sample. For the Orbitrap Exploris 480 MS runs, the loading amount was 500 ng. **c**, log-transformed ratios of quantified proteins. Scatter plots for all runs over the log-transformed protein intensities are displayed on the left, while density plots are on the right. Colored dashed lines represent expected log_2_(A/B) values for proteins from humans (green), yeast (orange) and *E. coli* (purple). Standard deviations are displayed on the density plots. FC_9, FC_4 and FC_1.5 are calculated from log_2_(E45H50Y5/E5H50Y45), log_2_(E40H50Y10/E10H50Y40) and log_2_(E30H50Y20/E20H50Y30), respectively. **d**, Number of precursors used and not used for protein quantifications in the MaxLFQ and QUANT2.0 algorithms. **e**, The intensity distribution of precursors used for protein quantifications in MaxLFQ and QUANT2.0 algorithms. **f**, Density plots of log-transformed protein ratios quantified using both the MaxLFQ and QUANT2.0 algorithms. FC, fold change. The unit in the density plots represents a proportion of the total distribution with a sum of 100 and is normalized such that the total sums up to 100 (%). Source data

To test the nDIA method performance for label-free quantification (LFQ), we analyzed mixed species proteome samples composed of human, yeast and *Escherichia* *coli* proteins in six different ratios (Fig. 3a). We analyzed the samples using nDIA (Supplementary Table 2) to benchmark the proteome coverage and quantitative accuracy and precision of the Orbitrap Astral mass spectrometer against DIA on an Orbitrap Exploris 480 mass spectrometer.

The Orbitrap Astral MS instrument identified >2×proteins and 3.5×peptide precursors in a 28-min LC gradient when analyzing 800 ng of HEK lysate compared with the Orbitrap Exploris 480 MS instrument with a 45-min LC gradient (Fig. 3b and Supplementary Fig. 6a). Notably, the Orbitrap Astral MS instrument demonstrated high technical reproducibility, with approximately 90% of proteins having CVs < 20% and low number (~3%) of missing values in technical triplicates (Supplementary Fig. 6b,c). Additionally, the MS/MS-based protein quantitation results in the Astral analyzer showed higher accuracy and lower standard deviations (~0.3) compared with the Orbitrap Exploris 480 MS instrument at the expected ratios for yeast and *E. coli* (Fig. 3c). Moreover, the higher protein and peptide coverage per protein resulted in improved protein quantification, particularly when using the MaxLFQ algorithm^29^. Unlike the QUANT2.0 algorithm, which only uses the top three peptide groups for protein quantification, the MaxLFQ algorithm utilizes over 99% of the measured precursors for protein quantification (Fig. 3d). The QUANT2.0 algorithm disregarded ~210,000 precursors, which were almost as abundant as the ~44,000 selected^30^ (Fig. 3e). Including these extra precursors in the MaxLFQ algorithm significantly improved quantification performance and demonstrates that nDIA provides accurate and precise protein quantification. To asses any possible abundance bias in quantitative precision and fold accuracy, we plotted the log_2_-tranformed protein intensities against the corresponding CVs of the protein ratios (Supplementary Fig. 7). Reassuringly, low-abundance proteins had higher fold-change CVs compared with high-intensity proteins. Density plots depicting fold-change CV distributions per species showed that approximately ~80% of the quantified proteins for *Homo sapiens* and *Saccharomyces cerevisiae* resulted in median CV below 10%, while for *E. coli* it was below 20%.

### Deep proteome acquisition through fast multi-shot strategy

**Fig. 4:**
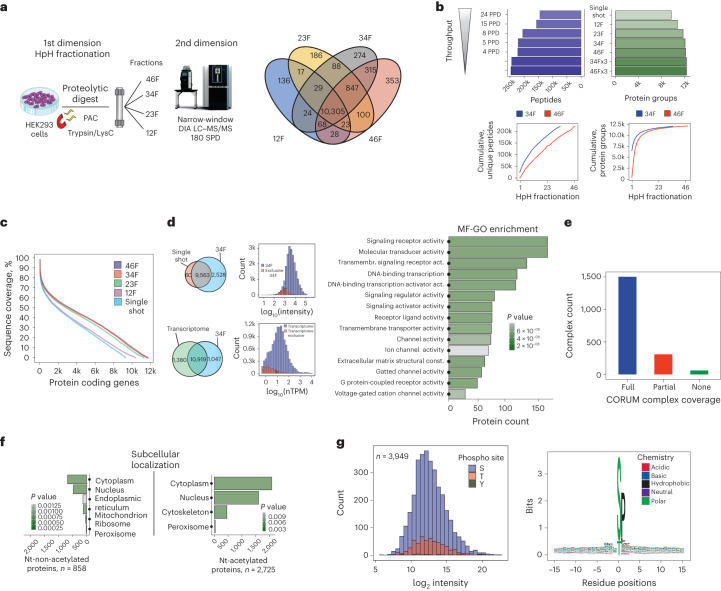
Comprehensive human proteomes by multi-shot proteomics. **a**, Experimental workflow of all HEK293 experiments and fractionation schemes (left). **b**, Protein and peptide identifications for different fractionation schemes and throughput are shown (top). Cumulative peptide identifications by fraction (bottom, left) and cumulative proteins (bottom, right) for both 46 and 34 fractionation schemes are presented. **c**, Comparison of sequence coverage achieved by different fractionation schemes and single-shot analysis. Overlap of protein groups identified between 34 fractionation scheme (5 proteomes per day (PPD)) and single-shot analysis (2-µg input and 30-min effective chromatographic gradient length). **d**, Protein group abundances in HEK293 identified in this dataset compared against single-shot analysis (left) are presented. GSEA functional enrichment analysis of the exclusively detected proteins in 34 fractionation scheme compared with single-shot analysis using gene ontology (GO) molecular function gene set. **e**, CORUM protein complex coverage of proteins identified in this dataset. **f**, Comparison of the GSEA functional enrichment analysis using GO cellular component terms gene set of Nt-acetylated and Nt-non-acetylated proteome. **g**, Abundance and sequence logo plot of detected phosphorylation sites without enrichment are shown. F, fraction; MF-GO, molecular function-gene ontology; Nt, N-terminal; nTPM, normalized transcripts per million. Source data

To overcome the inherent wide dynamic range of human protein abundances, a multi-shot proteomics strategy based on offline peptide fractionation can be employed to increase dynamic range and coverage compared with single-shot experiments. Fractionation decreases sample complexity but requires extensive MS measurement time. To maximize proteome coverage, we utilized high-resolution offline HpH reversed-phase peptide chromatography^31,32^ in combination with short online LC gradients and nDIA (2 Th) analysis on the Orbitrap Astral MS instrument. To identify the most effective strategy to comprehensively cover a human cell line proteome, we fractionated a tryptic digest of HEK293 cells into 46, 34, 23 or 12 HpH fractions (Fig. 4a).

By analyzing 200 ng from each of the 46 fractions with 5-min effective gradient DIA runs (6-h measurement time), we identified 12,179 proteins and 222,389 peptide sequences using Spectronaut (v.17) with directDIA+. This approach reduced the required MS acquisition time by sixfold and sample input by fivefold compared with previous approaches (Supplementary Fig. 8a). The quantitative performance in terms of reproducibility between different fractionation schemes was high, with Pearson correlation coefficients above 0.9 for all pairwise comparisons (Supplementary Fig. 8b). Additionally, ~90% of all quantified proteins in 23 HpH-fractionated HeLa and HEK cell lines had CVs < 10% (Supplementary Fig. 8c). Combining three biological replicates of each of the fractionation schemes resulted in a maximum coverage of 12,328 proteins from ~246,000 peptides from both 34 and 46 fractions (Fig. 4b). Notably, the 34 and 46 fractionation schemes achieved an average protein sequence coverage of ~40%.

Reducing the HpH fractionation scheme from 46 to 34 and 23 concatenated fractions resulted in similar proteome coverage of 99.4% and 96.7%, respectively (Fig. 4c). This suggests that routine acquisition of up to eight comprehensive human proteomes per day is feasible. The additional proteins identified by multi-shot compared with single-shot analysis are in the low-abundance range and represent important signaling proteins, including transmembrane receptors and transcription factors (Fig. 4d). The number of proteins quantified in the 34 fractionation scheme was comparable in coverage to next-generation RNA sequencing data of HEK293 (normalized transcripts per million > 0.5, 12,299 different gene products), with an overlap of 88.8% at gene level (Fig. 4d).

Our dataset encompasses ~80% of core protein complexes in the CORUM database^33,34^, providing evidence of its completeness (Fig. 4e). The extensive peptide coverage also enabled us to search for post-translational modifications, which typically require specific enrichment strategies before MS analysis^35^. We identified ~2,700 N-acetylation sites, which, as previously shown^4^, mainly target cytoplasmic and nuclear proteins (Fig. 4f). Additionally, we detected ~4,000 phosphorylation sites (site localization > 0.75; Supplementary Fig. 8d), covering the entire protein abundance distribution and likely representing the most abundant cellular sites (Fig. 4g). In accordance with previous studies^36–38^, phosphosites were mainly targets of proline-directed kinases such as the cyclin-dependent kinases and the mitogen-activated protein kinases.

### nDIA empowers systems biology and clinical proteomics

**Fig. 5:**
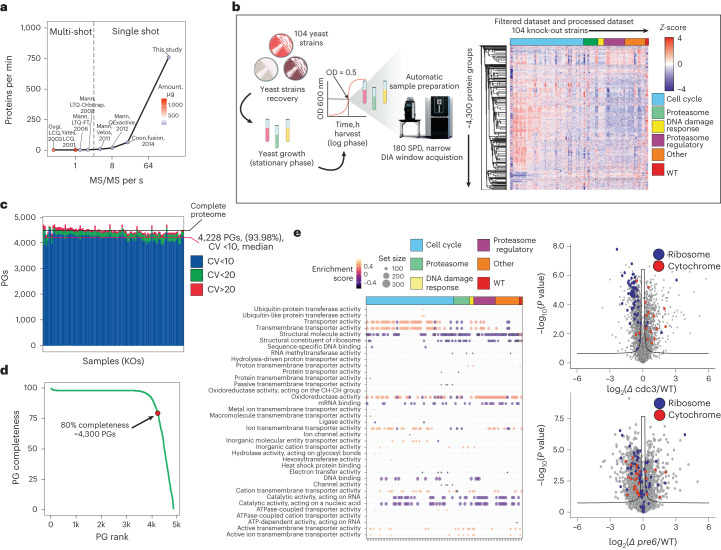
Functional genomics screen by comprehensive yeast proteome profiling. **a**, Rate of protein identifications as a function of mass spectrometer MS/MS scan rate for large-scale yeast proteome analysis (Yates, 2001 (ref. ^53^); Gygi, 2003 (ref. ^52^); Mann, 2006 (ref. ^35^); Mann, 2008 (ref. ^22^); Mann, 2011 (ref. ^54^); Mann, 2012 (ref. ^55^); Coon, 2014 (ref. ^23^)). **b**, Experimental setup for high-throughput yeast proteome MS analysis and dataset description. **c**, Protein identification numbers (mean) and CV per sample (*n* = 3). **d**, Data completeness curve showing the number of proteins quantified in a given percentage of samples. **e**, GSEA functional enrichment analysis results using GO molecular function terms gene set. Size of the dot indicates the set size and the color the normalized enrichment score (NES) (left). Volcano plot of differentially expressed proteins between the selected knockouts and a reference strain (right). *P* values were obtained by two-sided *t*-test and BH FDR corrected. BH, Benjamini–Hochberg; KO, knockout ; PG, protein group; WT, wild type. Source data

Functional genomics screens require large-scale approaches to evaluate gene functions, often through knockouts or knockdowns^39^. High-throughput proteomics can be used to analyze specific biological processes or diseases caused by genetic perturbations. Historically, the model organism to perform genome-wide screens in is *S. cerevisiae*, and consequently the proteomics community constantly strives to acquire comprehensive yeast proteomes in minimal MS analysis time (Fig. 5a). Given the essentially complete yeast proteome profiling provided by nDIA with 5-min gradients, we applied this to analyze a yeast strain library consisting of 104 gene knockouts targeting cell cycle, proteasome, DNA damage response and kinase genes (Fig. 5b). We consistently quantified ~4,500 proteins across all biological conditions and replicates, with 93.98% of proteins reproducibly quantified with CV < 10% (Fig. 5c).

In total, ~4,300 yeast proteins were consistently measured in 80% of samples (Fig. 5d). Analysis of knockout-regulated proteins showed that changes in protein abundances are linked to general biological processes such as cellular adaptation of translation rate and metabolic pathways (Fig. 5e). This dataset, collected in ~41.5 h and with great proteome depth for each strain, consistently reproduced the findings of a recent systematic yeast gene knockout screen^40^.

**Fig. 6:**
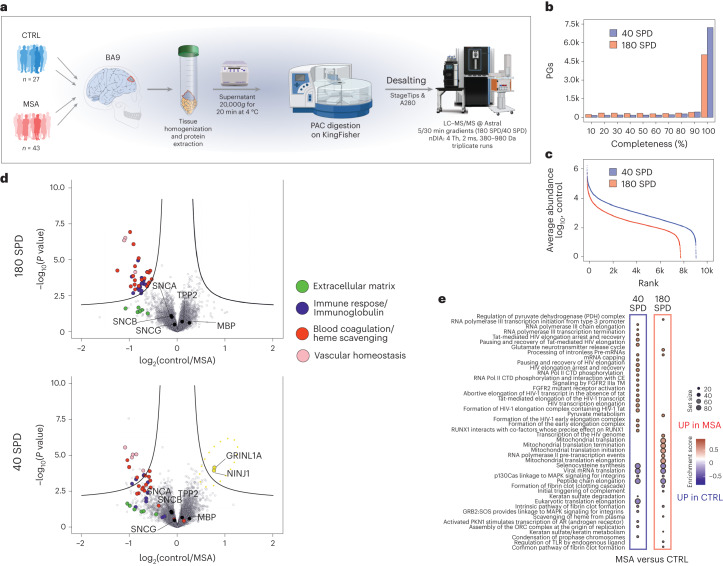
Enhanced high-throughput clinical proteomics. **a**, Sample preparation workflow of Broadmann’s area 9 (BA9) biopsies excised from either normal or MSA patient brains for proteome MS analysis. **b**, Histogram of data completeness showing the number of proteins quantified in a given percentage of samples for 180-SPD and 40-SPD methods. **c**, log_10_ abundance dynamic range detected by 180-SPD and 40-SPD methods. **d**, Volcano plots showing differential expression results between control and case samples (*P* values were obtained by two-sided *t*-test and BH FDR corrected) for both methods. **e**, GSEA functional enrichment analysis results using molecular pathways from the Reactome database. The top and bottom ten enriched molecular pathways for each method are shown. Size of the dot indicates the set size and the color the NES. PAC, protein aggregation capture. Source data

To demonstrate that nDIA is capable of achieving the high-throughput required for analyzing large clinical cohorts and conducting biomarker discovery experiments, we reanalyzed human brain protein extracts from Broadmann’s area 9 (BA9) from a multiple system atrophy (MSA) sample cohort of biopsies (*n* = 45 for MSA cases, and *n* = 29 for controls) in three technical replicates (222 LC–MS/MS runs) using the 180-SPD method (Fig. 6a). MSA is a rare and fatal neurodegenerative disease that affects most of the cortical brain areas^41^. Although the pathologic hallmark of MSA is accumulation of aggregated α-synuclein, the underlying pathophysiology of the disease explaining the origin of α-synuclein accumulation and the resulting loss of neurons remains unknown^42^. This fast nDIA analysis resulted in median coverage of 6,640 protein groups per brain sample, including more than 5,400 proteins quantified in >90% of samples. In contrast, analysis with half-an-hour active LC gradients using the Evosep One Whisper flow 40-SPD method enabled identification of 9,236 protein groups with a median of 8,320 proteins identified per sample and 7,239 proteins quantified in >90% of all samples (Fig. 6b). This comprehensive characterization of the MSA proteome was accomplished >20× faster with the 180-SPD and >5× faster with the 40-SPD method, resulting in 60% and 90% more proteins reproducibly quantified in at least 70% of the samples, respectively, compared with the original study^43^ (Supplementary Fig. 9a,b). The 180-SPD method quantified about 80% of the proteins quantified by 40 SPD (Fig. 6c). We were able to identify the same outliers as found originally, confirming that the status as an outlier is associated with the sample and not with the analysis method (Supplementary Fig. 9c). The nDIA analysis enabled us to reproduce the main biological findings reported in the original work, where proteins involved in blood coagulation and in the immune system (immunoglobulins and complement factors) were found with increased levels in the MSA brain parenchyma (Fig. 6d,e). The deeper proteome coverage with the 40-SPD method enabled the detection of proteins related to the glutamate release cycle and transcriptional processes, and these were highlighted through a gene set enrichment analysis (GSEA) to be elevated in the brain biopsies from patients with MSA (Fig. 6e). Altered glutamate metabolism has been proposed as a potential cause of MSA in animal models^44^, while disruption of transcription factor regulation has been related to the development of Parkinson-like symptoms^45,46^ (Supplementary Table 5). These results showcase the potential of nDIA to help identify biomarkers of biomedical importance with high throughput.

## Discussion

In the past decade, MS-based proteomics has advanced in MS data acquisition strategies and sample preparation methods, allowing high-throughput analysis of complex proteome samples^8,27,47–49^. This progress was due to technological advances, such as faster scanning mass spectrometers^10,50^. In this study, we introduce an MS acquisition approach based on nDIA at 200-Hz DIA MS/MS acquisition rate using the Orbitrap Astral mass spectrometer. This approach enables obtaining DDA-like DIA data, offering extensive proteome coverage and precise measurements at high speed, resolution and mass accuracy. Narrowing the isolation window in nDIA enhances selectivity similar to DDA methods, which was not possible with current state-of-art mass spectrometers. The nDIA strategy proved advantageous, notably in high-throughput proteome profiling experiments with short gradients, mitigating co-elution of multiple peptides and reducing chimeric spectra typically occurring in traditional DIA. Furthermore, as nDIA blurs the distinction between DIA and DDA, it creates opportunities for applications traditionally favoring DDA, such as unbiased peptidoform discovery via open-search strategies. Consequently, nDIA approaches could lead to implementation of commonly used DDA data processing strategies while addressing the missing value problem in DDA by leveraging the high proteome depth, data completeness and quantitative precision of nDIA workflows. For instance, the Orbitrap Astral mass spectrometer demonstrates the sensitivity needed to routinely acquire comprehensive proteomes using nDIA, maximizing peptide sequencing across the mass range, even with low ion loads. We optimized the acquisition speed of the Astral analyzer by scaling DIA isolation window size with maxIT, where a 2.5-ms maxIT results in >200-Hz MS/MS acquisition rate with 2-Th isolation windows for DIA. Moreover, when benchmarking the performance of nDIA against publicly available DIA-based reference datasets recorded on human cell lines using current state-of-the-art mass spectrometers (TripleTOF 6600, ZenoTOF 7600, timsTOF HT and Orbitrap Exploris 480), all with comparable gradient lengths and analyzed with the same software tools and settings, the Orbitrap Astral identified >3× more peptides and >2× protein groups in spectral library-free mode compared with any of the other MS platforms. Likewise, nDIA enables rapid acquisition of essentially complete human proteomes with a throughput of 5–8 comprehensive proteomes per day by using very short 5-min LC–MS/MS gradients to analyze the 23–34 HpH fractions. This strategy provides ten times higher throughput than previously, without compromising data quality or completeness. The higher peptide coverage resulting from this strategy enables direct identification of major post-translational modifications without specific enrichment, surpassing the capabilities of next-generation RNA sequencing^4^. We also demonstrated the advantage of nDIA for rapid systems biology studies by comprehensively quantifying 312 near-complete yeast proteomes in just 41.6 h. Finally, we showcased the potential of nDIA for rapid and in-depth acquisition of clinical samples with high protein coverage, reproducibility and robustness by analyzing a clinical cohort of MSA brain biopsies with 5-min active LC gradients. These results highlights the potential of nDIA to facilitate routine analysis of large clinical cohorts with the necessary depth and sample size to support clinical decision-making based on biomarker signatures.

A limitation of the nDIA approach in its current implementation is that it impacts overall sensitivity. Reducing the mass range with *n* number of narrow DIA windows reduces sensitivity proportionally by *n*-times. This means that when operating the Orbitrap Astral mass spectrometer at 200-Hz nDIA MS/MS acquisition rate, only 0.5% of the ion beam is sampled at any given time. A solution to this could be storing and sequential release of the precursors synchronized with the quadrupole mass filter to maintain sensitivity. Consequently, ion scheduling methods (for example, utilizing pre-separation by ion mobility) may offer feasible solutions in the future to further enhance sensitivity for nDIA. Alternatively, combining nDIA with optimal window placement for low-abundance targets in a hybrid-DIA^51^ approach or adding a component of retention-time, such as in dynamic DIA, could further improve the sensitivity and quantitative accuracy and precision. These achievements highlight the potential of MS-based proteomics for quantifying global proteome expression in human samples, including tumor-derived cell lines and clinical samples.

### Ethics declaration

This project was approved by the regional ethical committee of the Capitol Region (Denmark), j.nr. H-20015276, and carried out in accordance with the principles of the Declaration of Helsinki.

### Inclusion and ethics statement

We are committed to promoting diversity and inclusion in science and ensuring that our research is conducted ethically and responsibly. Our study was designed and conducted in accordance with ethical principles and guidelines, including obtaining informed consent from all participants and complying with relevant regulations and laws.

## Methods

### Sample preparation

#### Cell lines

Different human cell lines (HeLa (ATCC), HEK293 (ATCC)) were cultured in DMEM (Gibco, Invitrogen), supplemented with 10% FBS, 100 U ml^−1^ penicillin (Invitrogen) and 100 μg ml^−1^ streptomycin (Invitrogen), at 37 °C, in a humidified incubator with 5% CO_2_. Cells were collected at ∼80% confluence by washing twice with PBS (Gibco, Life technologies) and subsequently adding boiling lysis buffer (5% SDS, 5 mM Tris(2-carboxyethyl)phosphine, 10 mM chloroacetamide, 100 mM Tris, pH 8.5) directly to the plate. The cell lysate was collected by scraping the plate and then boiled for an additional 10 min, followed by micro-tip probe sonication (Vibra-Cell VCX130, Sonics) for 2 min with pulses of 1 s on and 1 s off at 80% amplitude. Protein concentration was estimated by BCA.

#### Yeast strains

The *Saccharomyces cerevisiae* strains used in this study were S288C isogenic yeast strains (MATα) wild-type, BY4743 and BY4743 yeast knockout collection from Horizon Discovery. Briefly, yeast was grown in YPD medium supplemented with G418 at 400 µg ml^−1^ at 30 °C. Overnight pre-culture was diluted to optical density (OD)_600_ = 0.2. Cultured yeast was collected at OD_600_ ~ 0.5 by centrifugation (10,000*g* for 5 min at 4 °C), washed two times with cold PBS and stored at −80 °C until use.

#### Tissue sampling

The human brains analyzed in the current study were donated to the Brain Bank at Bispebjerg-Frederiksberg Hospital (Copenhagen University Hospital, Denmark) or to the Medical Research Council (MRC) London Neurodegenerative Diseases Brain Bank (King’s College London, UK). All donated brains were neuropathologically examined to verify the diagnosis. In total, brains from 29 individuals showing no signs of neuropathological disease and 45 patients with MSA were included in the analysis (Supplementary Table 5 and Supplementary Table 2 in Rydbirk, Østergaard, Folke et al.^43^; one CTRL sample from the original study could not be included in the current study as there was no sample left). Collection and analysis of the samples were conducted in accordance with the World Medical Association Declaration of Helsinki and approved by the regional ethical committee of the Capitol Region (Denmark), j.nr. H-16025210 and H-15016232, and the Danish data protection agency (j.nr. P-2020-937). All Danish donors provided informed, written consent, whereas informed, written consent was provided either by British donors or by their next of kin. Brain tissue was stored at −80 °C until protein extraction. In brief, prefrontal cortex tissue was extracted by transferring ~100 mg of brain tissue from both gray and white matter to 500 μl of Tissue Extraction Reagent II (Invitrogen) containing 1% (v/v) Protease Inhibitor Cocktail (Sigma-Aldrich) in MagNA Lyser Green Beads tubes (Roche) on ice. The tissue samples were disrupted and homogenized at 6,000 r.p.m. for 25 s on a MagNA Lyser Instrument (Roche) with cooling for 90 s on an ice-rack between runs. Extracts were centrifuged at 20,000*g* for 20 min at 4 °C and supernatants were transferred to new tubes, aliquoted and stored at − 80 °C until analysis.

#### Preparation of samples for LC–MS/MS analysis

For global yeast proteome profiling, yeast cells were resuspended 1:2 in lysis buffer composed of 100 mM triethylammonium bicarbonate, pH 8.5, 5 mM Tris(2-carboxyethyl)phosphine hydrochloride, 10 mM chloroacetamide and 2% SDS. Cells were lysed by eight rounds of bead beating (1 min of beating, 1 min of rest, 66 Hz) in a Precellys 24 homogenizer with 400-µm silica beads (2:1, resuspended cells:silica beads). The extracted protein lysates were heated to 95 °C during 10 min, briefly sonicated and centrifuged at 16,000*g*, 4 °C. Afterwards, the protein concentration was approximated using the BCA assay (Pierce).

The yeast, brain extracts and human cell lines were digested overnight using the Protein Aggregation Capture protocol^49^ with the MagReSyn amine microparticles (ReSyn Biosciences). The proteolytic digestion was performed by addition of lysyl endopeptidase (LysC, Wako) and trypsin enzymes at 1:500 and 1:250 protein ratio, respectively. The samples were incubated at 37 °C overnight. The digestion was quenched by the addition of tri-fluoro acetic acid to a final concentration of 1%. Peptide mixtures from human cell lines were further concentrated on SepPaks (C_18_ Vac C_18_ Cartridge, 1 cc/50 mg, 55–105 μm, Waters). Final peptide concentration was estimated by measuring absorbance at 280 nm on a NanoDrop 2000C spectrophotometer (Thermo Fisher Scientific). The resulting peptide mixtures from yeast were desalted by Stage-tips, eluted and finally dried down using a SpeedVac vacuum concentrator. The protein digests from the mixed species for the LFQ analysis were purchased from Pierce for HeLa (88328), Promega for yeast (V7461) and Waters for *E. coli* (SKU: 186003196). They were mixed manually in six different ratios, E5-H50-Y45, E10-H50-Y40, E20-H50-Y30, E30-H50-Y20, E40-H50-Y10 and E45-H50-Y5, respectively. Samples were kept at −20 °C until further use.

### Offline HpH reversed-phase HPLC fractionation

HEK293 peptides (200 µg) were separated by HpH reversed-phase chromatography using a reversed-phase Acquity CSH C_18_ 1.7 μm × 1 mm × 150 mm column (Waters) on a Thermo Scientific UltiMate 3000 HPLC system (Thermo Fisher Scientific) with the Chromeleon software. The instrument was operated at a flow rate of 30 μl min^−1^ with buffer A (5 mM ABC) and buffer B (100% ACN). Peptides were separated by a multi-step gradient as follows: 0–10 min 6.5% B to 15% B, 10–59.5 min 15% B to 30% B, 59.5–67 min 30% B to 65% B, 67–70 min 65% B to 80% B, 70–77 min 80% B, 78–87 min 6.5% B. A total of 46 fractions were collected at 60-s intervals. Samples were acidified using 30 µl of 10% formic acid. Samples were dried down using a SpeedVac vacuum concentrator. Sample concatenation was performed manually afterwards to the following schemes: 34, 23 and 12 fractions. We injected 200 ng of each sample for LC–MS/MS analysis.

### LC–MS/MS analysis

LC–MS/MS analysis was performed on an Orbitrap Astral mass spectrometer coupled to a Thermo Scientific Vanquish Neo UHPLC or an Evosep ONE system, and interfaced online using an EASY-Spray source. Depending on the gradient used different set-ups were used, either trap-and-elute or direct injection into the column. Column type was also chosen according to the gradient employed (Supplementary Table 2). A blank run of the same gradient length was run after two or three runs.

For the DDA experiments, the Orbitrap Astral mass spectrometer was operated with a fixed cycle time of 0.5 s and with a full scan range of 380–980 *m*/*z* at a resolution of 180,000. The automatic gain control (AGC) was set to 500%. Precursor ion selection width was kept at 2-Th and peptide fragmentation was achieved by HCD (Normalized Collision Energy 30%). Fragment ion scans were recorded at a resolution of 80,000 and maximum fill time of 2.5 ms. Dynamic exclusion was enabled and set to 10 s.

For the DIA experiments, the Orbitrap Astral mass spectrometer was operated at a full-MS resolution of 180,000 or 240,000 with a full scan range of 380–980 *m/z* when stated. The full-MS AGC was set to 500%. Fragment ion scans were recorded at a resolution of 80,000 and maxIT of 2.5 ms. We used 300 windows of 2-Th scanning from 380 to 980 *m*/*z*, unless stated otherwise in Supplementary Table 2. The isolated ions were fragmented using HCD with 25% Normalized Collision Energy. For low-amount dilution series, the Orbitrap Astral mass spectrometer was connected to the FAIMS pro interface at compensation voltage −50 V. The full scan range was 400–800 *m*/*z* and the full-MS resolution was 240,000. Additional information can be found in Supplementary Tables 2 and 6. For the LFQ samples acquired in the Orbitrap Exploris 480 mass spectrometer, peptides were eluted online from the EvoTip using an Evosep One system (Evosep Biosystems) and analyzed at 30 SPD (45-min gradient) using a commercial 150-mm analytical column (EV1113 ENDURANCE COLUMN, Evosep Biosystems). The mass spectrometer was operated in positive mode using the DIA mode. Full scan precursor spectra (350–1,400 Da) were recorded in profile mode using a resolution of 120,000 at *m*/*z* 200, with a normalized AGC target of 300% and a maxIT of 45 ms. Fragment spectra were then recorded in profile mode, fragmenting 56 consecutive 13-Da windows (1 *m*/*z* overlap) covering the mass range 361–1,033 Da and using a resolution of 15,000. Isolated precursors were fragmented in the HCD cell using 27% normalized collision energy, a normalized AGC target of 1,000% and a maxIT of 22 ms.

Further details are described in Supplementary Tables 2 and 6.

### Raw MS data analysis

Raw files from DIA and DDA comparison experiments were analyzed in DIA-NN 1.8.1 (ref. ^56^) using an in silico DIA-NN predicted spectral library (4,299,848 precursors, allowing for C carbamidomethylation and N-terminal M excision and 1 missed cleavage). The spectral library was generated from a human reference database (UniProt 2022 release, 20,598 sequences). The DIA-NN search included the following settings: Protein inference = ‘Genes’, Neural network classifier = ‘Single-pass mode’, Quantification strategy = ‘Robust LC (high precision)’, Cross-run normalization = ‘RT-dependent’, Library Generation = ‘IDs, RT and IM Profiling’ and Speed and RAM usage = ‘Optimal results’. Mass accuracy and MS1 accuracy were set to 0 for automatic inference. ‘No share spectra’, ‘Heuristic protein inference’ and ‘MBR’ were checked. The MS1 quantification for DDA approach was performed as follows: The output results from DIA-NN were filtered for PG.Qvalue < 0.05, Q.Value < 0.01 and Global.PG.Q.Value < 0.01, then the DIA-NN R package was used to calculate the MaxLFQ abundance for protein groups. MaxLFQ abundance was calculated based on ‘MS1.area’ column prior normalization by the ratio Precursor.Normalized/Precursor.Quantity. DIA approach was assessed using MS2-centric methods from DIA-NN output.

Raw files from single-shot dilution series of HEK peptides, window optimization and clinical samples were analyzed in Spectronaut v.17 (Biognosys) with a library-free approach (directDIA+) using the human reference database (UniProt 2022 release, 20,598 sequences) complemented with common contaminants (246 sequences). Cysteine carbamylation was set as a fixed modification, whereas methionine oxidation and protein N-terminal acetylation were set as variable modifications. Precursor filtering was set as *Q* value, and cross-run normalization was unchecked. Each experiment was analyzed separately, and those that contained different experimental conditions (different input amounts or acquisition methods) were searched, enabling method evaluation and indicating the different conditions (each one with *n* = 3 experimental replicates) in the condition setup tab.

Raw files from single-shot analysis of different gradients and low-amount dilution series (50 pg to 10 ng) were analyzed in Spectronaut v.18 (Biognosys) with a library-free approach (directDIA+) using the human reference database (UniProt 2022 release, 20,598 sequences) complemented with common contaminants (246 sequences). Cysteine carbamylation was set as a fixed modification, whereas methionine oxidation and protein N-terminal acetylation were set as variable modifications. Precursor filtering was set as *Q* value, and cross-run normalization was unchecked. Each gradient was analyzed separately, and each analysis contained three experimental replicates. For low-amount dilution series the quantification was performed at Orbitrap MS1 level.

Raw files from LFQ analysis of the mixed species samples were analyzed in Spectronaut v.17 (Biognosys) with a library-free approach (directDIA+) using a benchmark reference database for the three species (31,657 sequences in total). Cysteine carbamylation was set as a fixed modification, whereas methionine oxidation and protein N-terminal acetylation were set as variable modifications. Precursor filtering was set as *Q* value, and cross-run normalization was enabled. Each experiment consisting of samples with the same loading amounts was analyzed separately, and each condition contained three experimental replicates.

Raw files from different fractionation schemes were analyzed in Spectronaut v.17 (Biognosys) with a library-free approach (directDIA+) using the human reference database (UniProt 2022 release, 2,058 sequences) complemented with common contaminants (246 sequences). Methionine oxidation and protein N-terminal acetylation were set as variables, whereas cysteine carbamylation was set as a fixed modification. Precursor filtering was set as *Q* value. Each fractionation scheme was searched independently, except for searches performed in triplicate. Quantification was performed using the MaxLFQ algorithm embedded in iq R package^57^. Briefly, extended Spectronaut output results were filtered as follow: PG.Qvalue < 0.01 and EG.Qvalue < 0.01. Then, the MaxLFQ algorithm was applied using PG.Genes and PG.ProteinNames for protein annotation. Finally, to determine the percentages of residues in each identified protein sequence (sequence coverage), the program Protein Coverage Summarizer was used. The human reference database used for quantification and a file containing all detected peptide sequences with a protein name associated (PG.Qvalue < 0.01 and EG.Qvalue < 0.01) were utilized for protein assembly and sequence coverage calculation. For the phosphopetides search, the 34 fraction scheme was analyzed in triplicate using an empirical library generated in-house by the HpH fractionation (12 fractions) of phosphopeptide enrichment (119,793 precursors). Spectronaut output was reformatted using the Perseus plugin peptide collapse^58^ to create a MaxQuant-like site-table.

Yeast knockout collection raw files were analyzed using Spectronaut v.17 (Biognosys) with a library-free approach (directDIA+) using a database composed of the canonical isoforms of *S. cerevisiae* (6,059 sequences) complemented with a common contaminant database (246 sequences). Briefly, cysteine carbamylation was set as a fixed modification, whereas methionine oxidation and protein N-terminal acetylation were set as variable modifications. Precursor filtering was set as *Q* value, and cross-run normalization was checked.

In Spectronaut (v.17 and v.18), protein grouping was performed using the default protein inference workflow with IDpicker as the inference algorithm.

Plots from Fig. 1b–d were based on MS1 feature detection output retrieved by MaxQuant (v.1.6.7.0). Total ion current intensity for Fig. 3c was obtained from MaxQuant (v.1.6.14.0). Representative raw files for each method were loaded into MaxQuant and analyzed without the indication of a FASTA file, to extract only the relevant MS features.

Enrichment analysis was performed with the package clusterProfiler^59^ and using the org.Sc.sgd.db and org.Hs.eg.db. All data analysis was performed using R v.4.2.2 and R studio v.2022.12.0 Build 353.

### Reporting summary

Further information on research design is available in the Nature Portfolio Reporting Summary linked to this article.

## Online content

Any methods, additional references, Nature Portfolio reporting summaries, source data, extended data, supplementary information, acknowledgements, peer review information; details of author contributions and competing interests; and statements of data and code availability are available at 10.1038/s41587-023-02099-7.

## Supplementary information

Supplementary Information Supplementary Figs. 1–9 and Notes 1 and 2.

Reporting Summary

Supplementary Table 1 MS method overview.

Supplementary Table 2 60-min gradient protein group numbers for different dilutions.

Supplementary Table 3 Identification numbers for different gradient lengths.

Supplementary Table 4 Identification numbers for low-range amounts.

Supplementary Table 5 Protein groups identification numbers for MSA clinical cohort.

Supplementary Table 6 Experimental overview.

## Source data

Source Data Fig. 1 Statistical source data.

Source Data Fig. 2 Statistical source data.

Source Data Fig. 3 Statistical source data.

Source Data Fig. 4 Statistical source data.

Source Data Fig. 5 Statistical source data.

Source Data Fig. 6 Statistical source data.

Source Data Extended Data Fig. 1 Statistical source data.

## Data Availability

Data are available via the ProteomeXchange with the following identifiers: (1) DDA versus DIA comparison: PXD046453; (2) fractionation strategies: PXD046372; (3) three-species mix: PXD046444; (4) yeast KO collection: PXD046386; (5) clinical samples (MSA versus Ctrl): PXD046417; (6) single-cell data: PXD046357; (7) single-shot dilution series: PXD046283; and (8) window optimization: PXD046285. Supplementary Table 6 contains an overview of the experiments. Source data are provided with this paper.
