# Supplementary material for: Ultra-fast label-free quantification and comprehensive proteome coverage with narrow-window data-independent acquisition
Source: Nat Biotechnol. 2024 Feb 1;42(12):1855–66. doi: 10.1038/s41587-023-02099-7 (PMC11631760; doi:10.1038/s41587-023-02099-7)
Supplement: Supplementary file 1 — Supplementary Figs. 1–9 and Notes 1 and 2. [file 41587_2023_2099_MOESM1_ESM.pdf]

nature biotechnology

Article

https://doi.org/10.1038/s41587-023-02099-7

Ultra-fast label-free quantification and comprehensive proteome coverage with narrow-window data-independent acquisition

In the format provided by the authors and unedited

Supplementary material

Supplementary Figure1

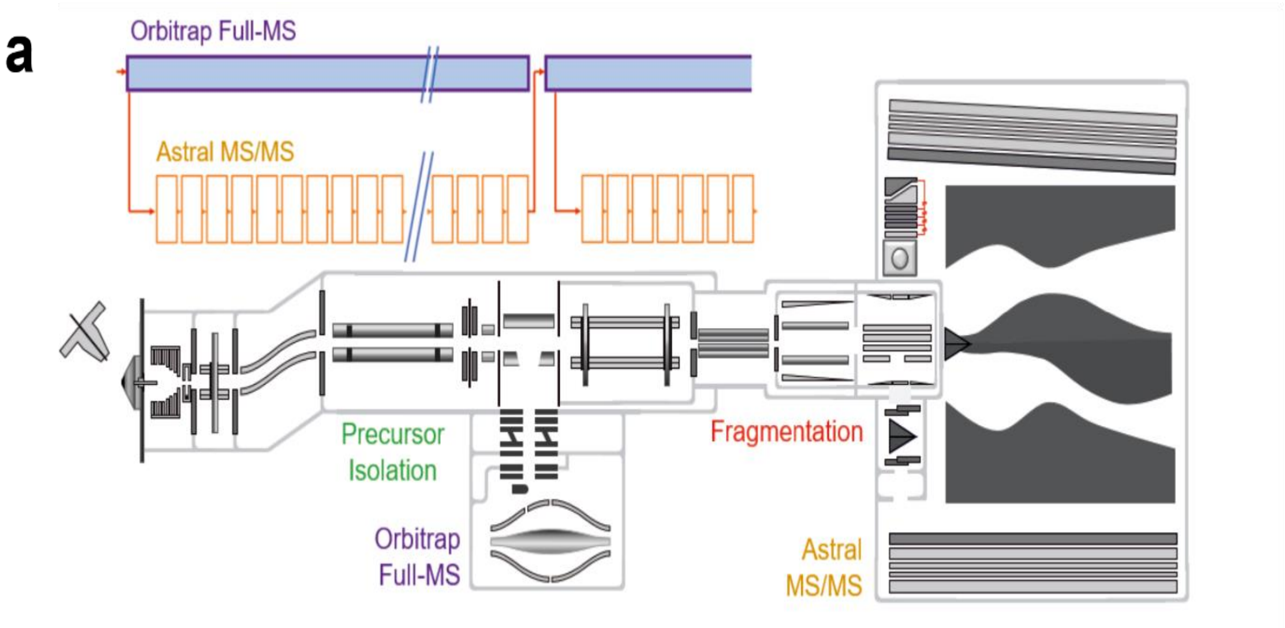

Supplementary Figure1. Schematics of a Quadrupole Orbitrap Exploris 480 mass spectrometer coupled to the Asymmetric track lossless mass analyzer (Astral).

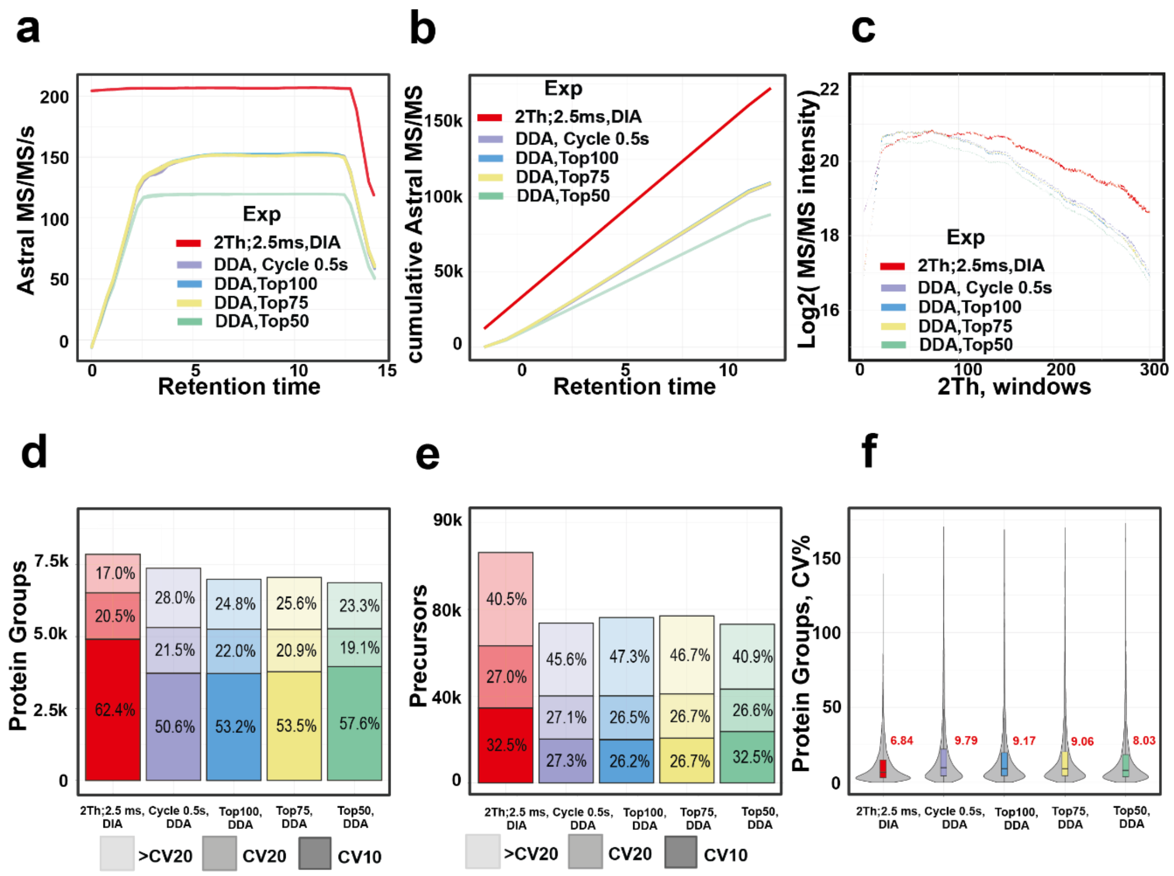

Supplementary Figure 2. Comparative analysis of different DDA methods compared to fast scanning nDIA in 15-minute gradients. (a) MS/MS scan rate over the 15 min chromatographic gradient length. nDIA fast (2.5 ms) and DDA(cycle 0.5, top 100, 75 and 50) methods are depicted by different colors (red, purple, blue, yellow and green respectively) (b) Cumulative MS/MS scans across 15 min chromatographic gradient acquired in Astral analyzer. (c) Log 2 intensity of MS/MS spectra measured in the Astral analyzer through the m/z range (300 2Th windows; 380-980) with DDA and DIA (d) Quantified protein groups measured with DDA and nDIA operation mode below 10 and 20 % of coefficient of variation (CV) (n=3, 200 ng tryptic HeLa peptides). (e) Quantified and identified precursors below 10 and 20 % of coefficient of variation (CV) with DDA and nDIA approaches (n=3, 200 ng tryptic HeLa peptides). (f) Coefficients of variation (CVs, %) for quantified protein groups by different operation modes. Median CV is shown in red for each approach (n=3, 200 ng tryptic HeLa peptides). Box limits indicate the 25th and 75th percentiles as determined by R software; whiskers extend 1.5 times the interquartile range from the 25th and 75th percentiles, outliers are represented by dots.

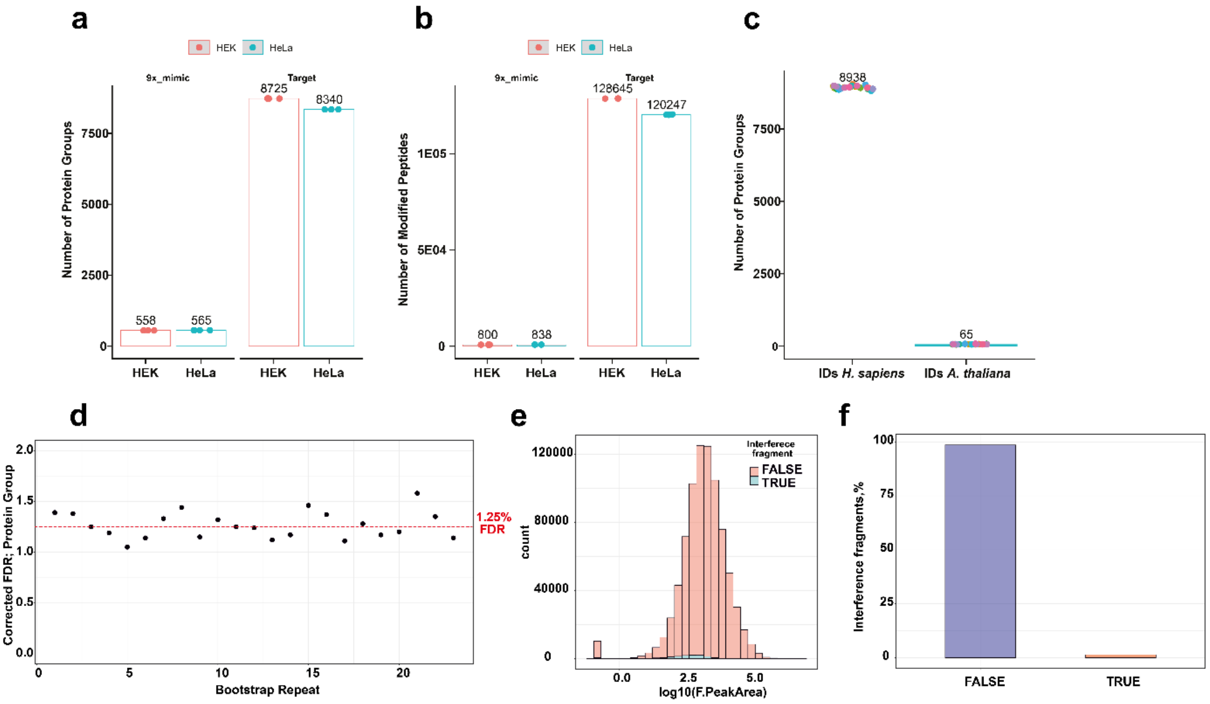

Supplementary Figure 3 | Entrapment sequence analysis to evaluate the false discovery rate (FDR) in Orbitrap Astral. (a-b) Number of proteins (a) and modified peptides (b) identified in target database and a decoy mimic database. The mimic database approach shuffles the target database 9 times and appends the proteins flagged as “mimic”. It keeps the original amino acid composition from the target human protein sequence database. (c) Number of proteins identified from a bootstrap empirical FDR calculation experiment. A protein sequence database file from Arabidopsis thaliana was combined with the human protein sequence database for the search process. A total of 23 repetitions were carried out in the bootstrap analysis. (d) Corrected FDR calculated by 23 iterations. (e) Distribution of fragment peak area used for quantification. (f) Percentage of interference fragments used for quantitation detected by SN v.18.

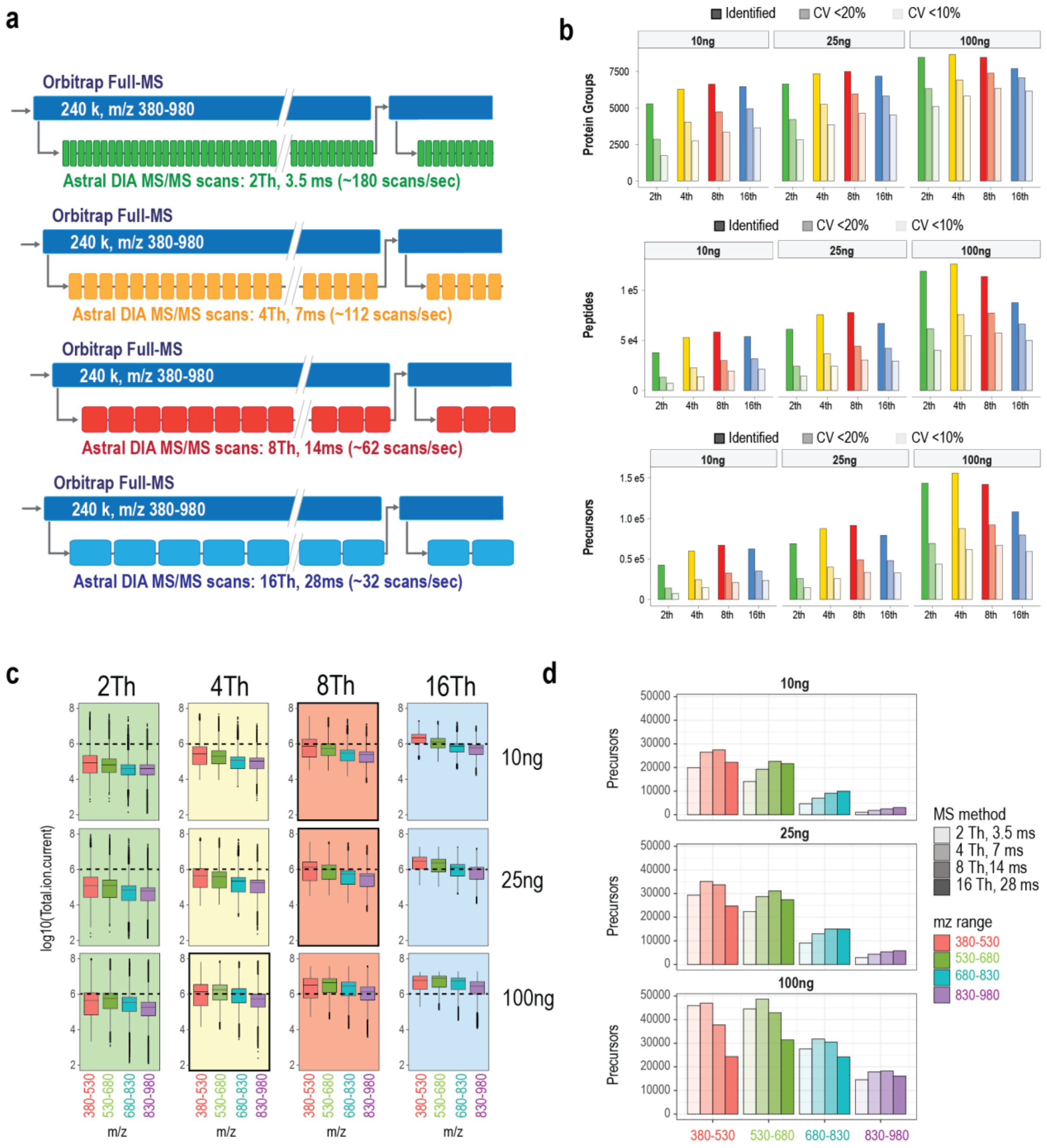

Supplementary Figure 4. Optimization of DIA acquisition parameters for low peptide loads (a) Schematic representation of acquisition cycles in the Orbitrap-Astral analyzers with different maximum IT and window width parameters. Orbitrap MS1 scans were acquired at 240K while in parallel Astral scans were acquired during a duty cycle of 1 second. For each method, the injection time was scaled to the window width, so the same mass range is covered in each cycle: 2Th and 3.5ms, 4Th and 7ms, 8Th and 14ms, 16Th and 28ms. (b) Protein groups, precursors and peptides (average of n=3) identified for 10, 25 and 100 ng

using different window widths, as depicted in panel A. Next to each bar the number of protein groups, precursors and peptides quantified with a CV<20% and CV<10% (c) Boxplots of total ion current measured at MS2 level, grouped in four mass ranges (380-530, 530-680, 680-830, 830-980), for different sample loadings and different acquisition methods. Highlighted, the method that gives higher identifications for that sample load. Black dashed line indicates the highest median intensity obtained for 100 ng and 4Th, which gives the highest identification rate (n=3). (d) Precursors (average of 3 technical replicates) identified in four mass ranges (380-530, 530-680, 680-830, 830-980) using different acquisition methods (injections times and window width). Box limits indicate the 25th and 75th percentiles as determined by R software; whiskers extend 1.5 times the interquartile range from the 25th and 75th percentiles, outliers are represented by dots.

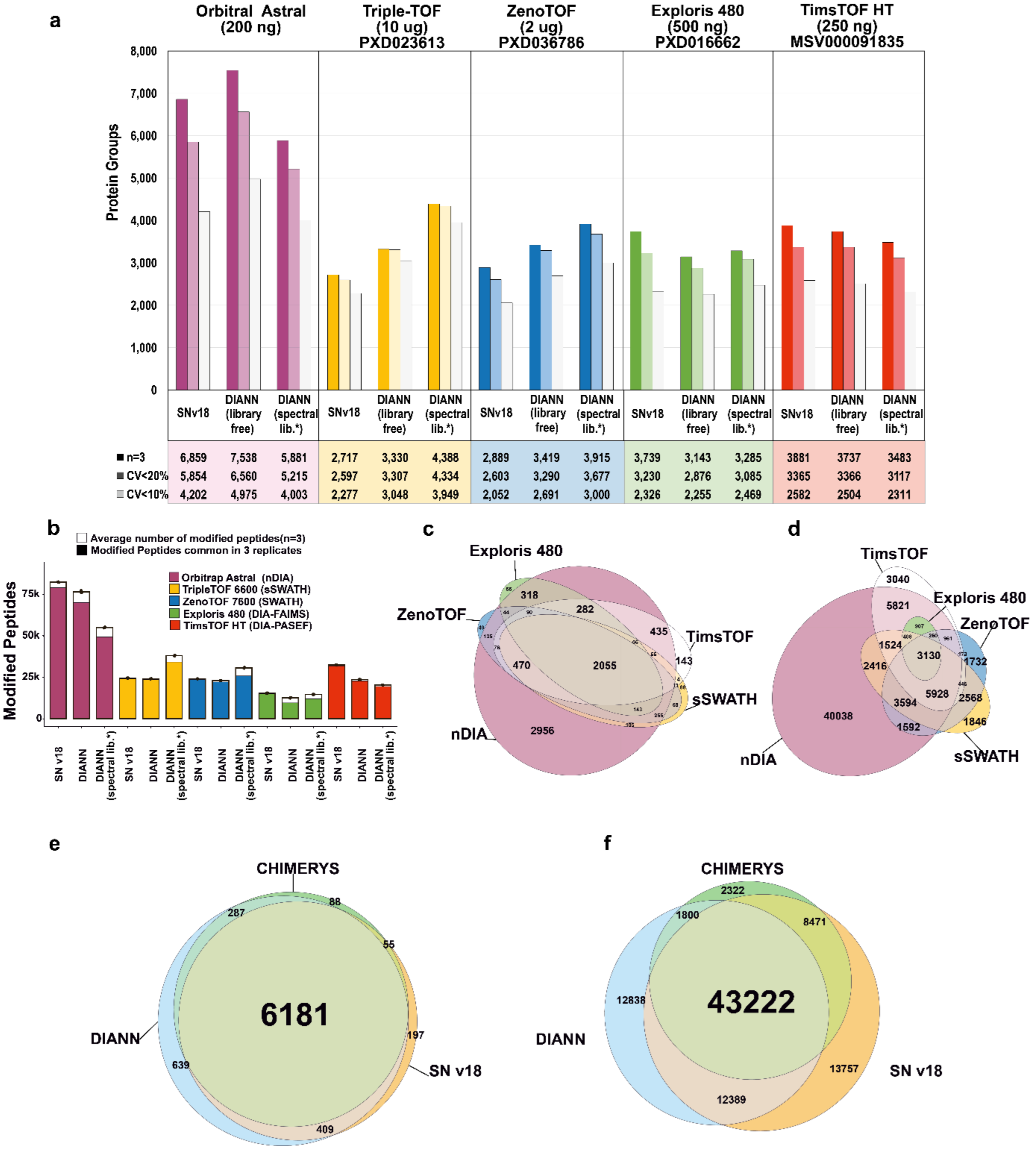

Supplementary Figure 5. Comparative analysis of the performance of state-of-art MS instruments with five-minute gradients. (a) Protein groups identification in four different MS instruments using directDIA in Spectronaut (SN) V18 or DIA-NN V1.8.1 in spectral library free mode or with a spectral library. First bar represents the number of protein groups found in three technical replicates, the second bar those with CV<20% and the third bar those with CV<10%. * Gas phase fractionation spectral library from PXD023613. ** 5minute gradient spectral library from PXD016662. (b) Bar plot of number of modified peptide sequences

identified in the different MS instruments with the search strategies from panel a. In dark, number of modified peptides identified in three technical replicates. In white, average number of modified peptides in three technical replicates. (c) Euler diagram of protein groups identified in three technical replicates using DIA-NN in library free mode in four MS platforms. (d) Euler diagram of modified peptides identified in three technical replicates using DIA-NN in library free mode in four MS platforms. (e) Euler diagram of protein groups identified in three technical replicates in the Orbitrap Astral in DIA-NN (library free), in Spectronaut (directDIA+) and in Chimerys. (f) Euler diagram of peptides identified in three technical replicates in the Orbitrap Astral MS in DIA-NN (library free), in Spectronaut (directDIA+) and in Chimerys. nDIA: narrow window DIA, sSWATH: scanning SWATH.

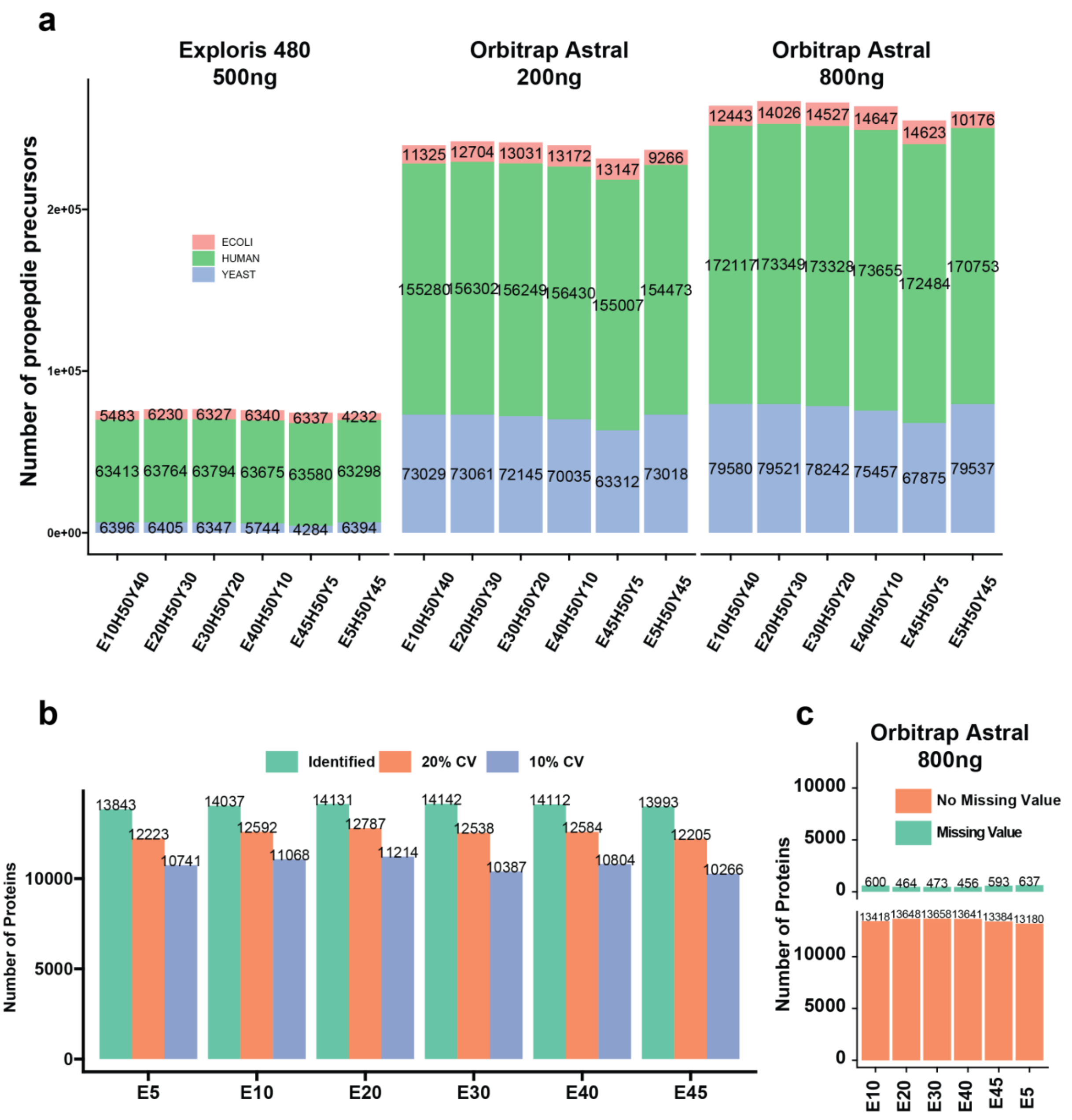

Supplementary Figure 6. Protein, peptides and precursor numbers and reproducibility. (a) Peptide and precursor numbers per species and sample. (b) Proteins identified and quantified below 10% and 20% coefficient of variation (n=3) (c) Data completeness per condition. Data presented in b and c is derived form 800 ng input experiments.

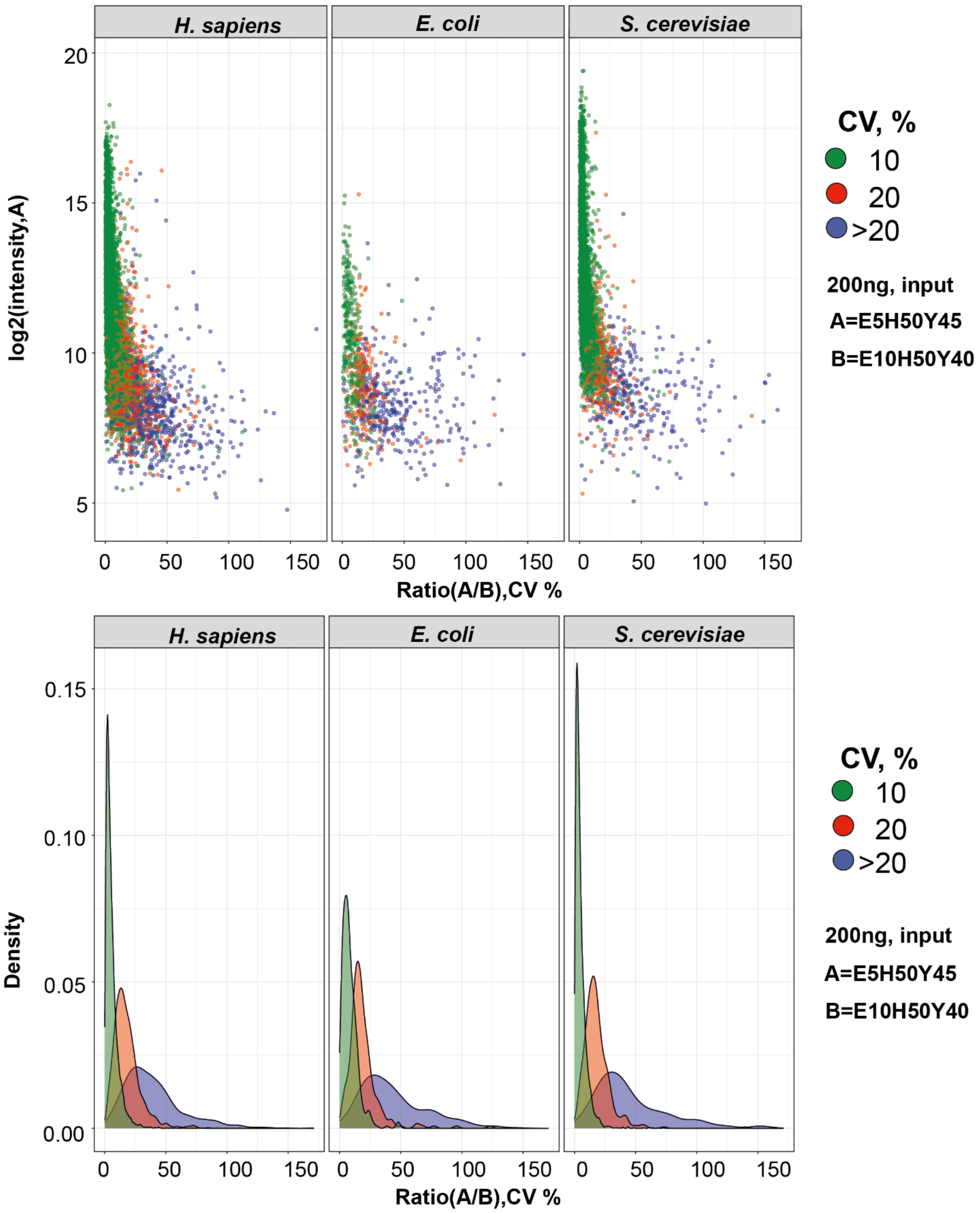

Supplementary Figure 7. Quantitative precision and fold accuracy on Orbitrap Astral MS. (a) Scatterplot depicting the log2 (A, intensity) as function of the fold change coefficient of variation of log2(A/B). Log-transformed ratios A/B and log-transformed intensity of quantified proteins are shown. The upper panel of the figure displays scatterplots of log-transformed protein intensities

versus fold change CV for each species, while the bottom panel shows density plots that illustrate the distribution of fold change CV by species.

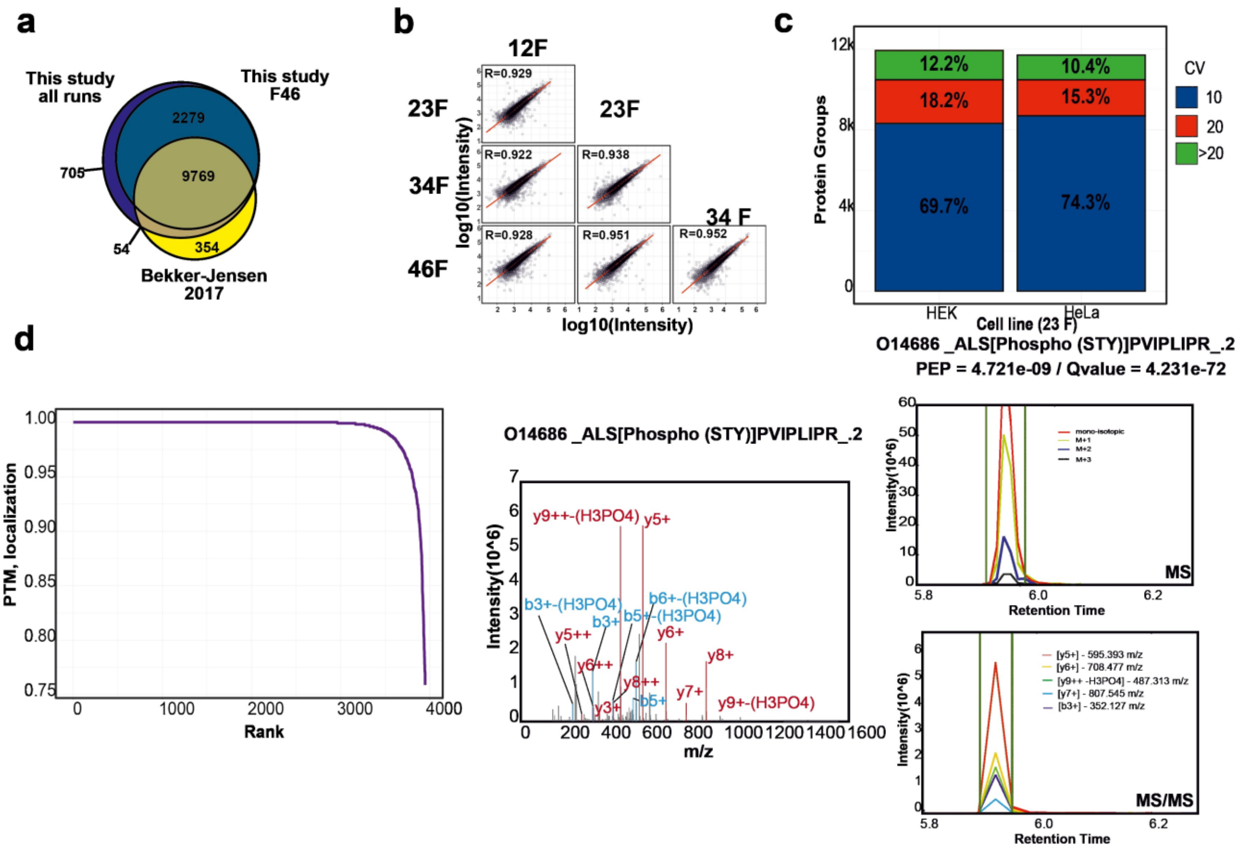

Supplementary Figure 8. Multi-shot proteomics approach reproducibility (a) Overlap of protein-coding genes identified in this HEK293 dataset against the state-of-the-art published dataset. (b) Quantitative reproducibility of the method. (c) Protein group coefficient of variation for 23 fractionated cell . HeK and HeLa cell lines. (d) PTM. Localization as function of PTM. Localization ranked phosphopeptides identified in a 34 fractioned HEK293 tryptic peptide sample (left). MS/MS spectrum (right) and extracted ion chromatogram (top MS1, bottom MS2) of the phosphopeptide ALS(p)PVIPLIPR (2+) found in a 34 fractionated HEK293 peptide sample.

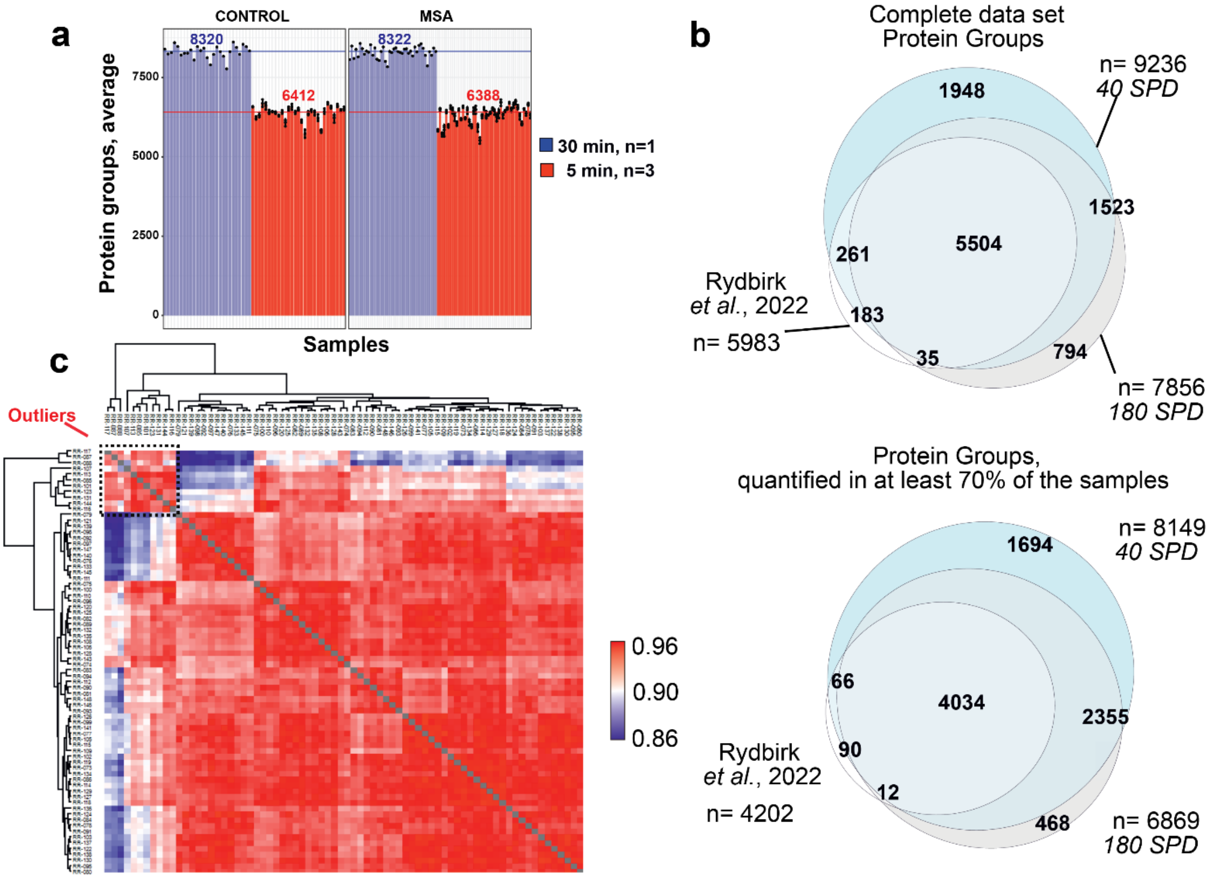

Supplementary Figure 9. Multiple system atrophy (MSA) vs Control data set description. (a) Protein identification ids (mean) with standard-deviation error bars. Black dashed line depicts the median number of identification found in the whole dataset (n=69 and 74 biological independent samples for 30 min and 5 min gradients respectively). (b) Proportional Venn diagrams depicts the overlap of protein groups identified between both datasets (original study[1] and new dataset). Upper: No filtered data sets are compared. Bottom: Filtered dataset for proteins quantified in at least 70% of the samples. (c) Heatmap of the correlation matrix generated by the Pearson r correlation coefficient for all the samples.

Supplementary Note1

Both Orbitrap^{TM} and conventional continuous-beam time-of-flight (ToF) analyzers suffer notable impediments. The Orbitrap instruments benefit from very high 30-50% ion transmission and almost 100% duty cycle by accumulating ions in the coupled pulsed extraction C-Trap prior to analysis[2].However, ion current detection requires multiple ions of the same species to produce identifiable signal, which favors longer injection times and transient lengths making faster acquisition less sensitive. In contrast, ToF analyzers employ electron multipliers that are single ion sensitive, but typically have poor ion transmission to the detector caused by ion losses within the orthogonal ion injectors and by multiple grids within the analyzer[3]. ToF analyzers typically operate at kHz frequencies, but require many averaged scans to accumulate sufficient ion signal for a high-quality spectrum. The employment of upstream trapped ion mobility devices in ToF mass spectrometers has seen notable success for reduction in quadrupole isolation losses by presenting a narrowed m/z range to the quadrupole in synchronization with ion isolation in it, which compensates somewhat for ion losses at the ToF analyzer[4].

The Astral analyzer itself represents an open electrostatic trap with destructive ion detection, and is formed of two opposing, but slightly converging, elongated ion mirrors, with a set of shaped “ion foil” electrodes mounted between them9. Injected ions are focused in the plane of the analyzer by a pair of lenses, and given appropriate injection angle by prism-shaped deflectors. The ions oscillate between the mirrors while drifting down the mirror channel. With each pass through the angled mirrors, the trajectory is deflected in a similar manner to light, until the drift velocity is completely negated and then reversed, causing the ions to pass back up

the analyzer to a high dynamic range detector. Particularly notable about this action is that the ion packet is allowed to diffuse into a wide spatial distribution, suitable for minimizing charge density and consequent space charge effects, before being refocused on its return path. As ions are accumulated and injected at 200Hz, rather than conventional low efficiency sampling at kHz rates, it is crucial that the analyzer be tolerant to tens of thousands of ions within a single shot.

The ion foil electrodes, mounted above and below the ion path, serve to both compensate for the flight time aberration induced by the mirror convergence, and also improve the energy acceptance of the drift reflection and thus quality of spatial focus at the detector. Combined with the use of gridless mirrors, incorporating a lens electrode to stabilize ion motion out of the plane of the analyzer, this makes for a near-lossless ion transfer, key to achieving higher ion transmission than the Orbitrap analyzer[9]. The overall flight path exceeds 30m, or 24 oscillations between the mirrors, and ions arrive at the detector with nanosecond scale focusing, resulting in high >80K resolving power, unattainable in a conventionally arranged and scaled time-of-flight analyzer without substantial cutting of the ion beam. The acquisition time of the Astral analyzer is typically set to 2 ms, still much faster than the 5ms instrument cycle time, which is limited primarily by the ion processor operation and the ion accumulation time required to build up sufficient ions for analysis.

Also of importance is to note the high dynamic range detector. The system's very high ion transmission means that individual m/z ion packets may easily incorporate 1000 ions per shot, whilst conventional detector systems have a linear range of <2 orders of magnitude, Here ions are post-accelerated to 14keV before conversion to electrons via conversion dynode, transmitted and accelerated to a scintillator, and

then converted to light and detected by a photomultiplier tube PMT. The PMT output is split into two and fed into preamplifier channels with a 10x difference in gain, and each channel separately recorded as digital signal. Saturated peaks from the high gain channel may then be discarded and replaced with low gain channel counterpart data, creating a 10x dynamic range improvement vs regular single-channel detection. The detector gain is calibrated so that single ions are detected above a 6-sigma noise threshold with 80% efficiency. Multiple-ion peak widths are much broader than single ions, and broaden further under space charge towards 1000 ions in peak, which reduces resolution but has a silver lining of reducing peak height and thus expanding dynamic range further, while statistical mass accuracy remains high. As the PMT output is grounded, capacitive coupling is not required and post-peak ringing artifacts eliminated, resulting in reduced requirements on PTM gain and wider dynamic range.

Whilst the intra-scan linear dynamic range is 3-4 orders of magnitude, with a dependency on m/z related peak width, the inter-scan dynamic range is extended by the ability to automatically vary ion accumulation time (automatic gain control AGC) to prevent overload, just as employed in Orbitrap-based instruments. In a typical experiment, this may be varied between 0.03 and 3 ms, adding a further 2 orders of magnitude to the dynamic range. Longer maximum accumulation times may be employed, but at the cost of reducing the repetition rate.

The sum of these technologies is a mass analyzer that may operate at 200 Hz, with each single shot producing a well-populated, high resolution accurate mass spectrum with no need to undergo averaging. The device combines linear ion trap-

like ion sensitivity, with ToF-like maximum acquisition rates and ion transmission exceeding that of even Orbitrap analyzers.

Supplementary Note 2

The reproducibility and depth (median CV < 3 % and 9,619 proteins) achieved by the 28-min gradient analysis (Extended Data Fig.1b-c) could be explained by the enhanced MS signal from using shorter gradients (28 min vs 60 min), enabling increased scanning speed (>200 Hz) by reducing the injection time from 3.5 ms to 2.5 ms for the same injection load. This enables similar protein and peptide coverage in half the acquisition time without sacrificing sensitivity (Extended Data Fig1b). With a 200-ng and 12.5 min gradient, we detected up to 8120 proteins with median CVs < 6% or ~8400 proteins with median CV<10% in half-an-hour using 1µg (Extended Data Fig. 1b-c) (Supplementary Table S2). The 200-ng loading with a 5-min gradient reached 6828 proteins (Extended Data Fig. 1b) (Supplementary Table S2), with 3500 covered within the first minute of peptide elution (Extended Data Fig.1d). The high identification rate of ~11,000 peptides/min or >1000 proteins/min means that even with narrow window 2-Th DIA scans, chimeric spectra can be deconvoluted by commonly used DIA software (Extended Data Fig.1e).

References

1. Rydbirk, R. *et al.* Brain proteome profiling implicates the complement and coagulation cascade in multiple system atrophy brain pathology. *Cellular and Molecular Life Sciences* **79**, (2022).
2. Makarov, A. *et al.* Performance evaluation of a hybrid linear ion trap/orbitrap mass spectrometer. *Anal Chem* **78**, 2113–2120 (2006).

3. Chernushevich, I. V., Loboda, A. V. & Thomson, B. A. An introduction to quadrupole-time-of-flight mass spectrometry. *Journal of Mass Spectrometry* **36**, 849–865 (2001).
4. Skowronek, P. *et al.* Synchro-PASEF Allows Precursor-Specific Fragment Ion Extraction and Interference Removal in Data-Independent Acquisition. *Mol Cell Proteomics* **22**, (2023).
