# Supplementary material for: Ultra-fast label-free quantification and comprehensive proteome coverage with narrow-window data-independent acquisition
Source: Nat Biotechnol. 2024 Feb 1;42(12):1855–66. doi: 10.1038/s41587-023-02099-7 (PMC11631760; doi:10.1038/s41587-023-02099-7)
Supplement: Supplementary file 2 — Reporting Summary [file 41587_2023_2099_MOESM2_ESM.pdf]

Corresponding author(s): Jesper V. Olsen

Last updated by author(s): Oct 27, 2023

Reporting Summary

Nature Portfolio wishes to improve the reproducibility of the work that we publish. This form provides structure for consistency and transparency in reporting. For further information on Nature Portfolio policies, see our Editorial Policies and the Editorial Policy Checklist.

Statistics

For all statistical analyses, confirm that the following items are present in the figure legend, table legend, main text, or Methods section.

n/a	Confirmed	
☐	☒	The exact sample size (*n*) for each experimental group/condition, given as a discrete number and unit of measurement
☐	☒	A statement on whether measurements were taken from distinct samples or whether the same sample was measured repeatedly
☐	☒	The statistical test(s) used AND whether they are one- or two-sided<br>*Only common tests should be described solely by name; describe more complex techniques in the Methods section.*
☐	☒	A description of all covariates tested
☐	☒	A description of any assumptions or corrections, such as tests of normality and adjustment for multiple comparisons
☐	☒	A full description of the statistical parameters including central tendency (e.g. means) or other basic estimates (e.g. regression coefficient) AND variation (e.g. standard deviation) or associated estimates of uncertainty (e.g. confidence intervals)
☐	☒	For null hypothesis testing, the test statistic (e.g. *F*, *t*, *r*) with confidence intervals, effect sizes, degrees of freedom and *P* value noted<br>*Give P values as exact values whenever suitable.*
☒	☐	For Bayesian analysis, information on the choice of priors and Markov chain Monte Carlo settings
☐	☒	For hierarchical and complex designs, identification of the appropriate level for tests and full reporting of outcomes
☐	☒	Estimates of effect sizes (e.g. Cohen's *d*, Pearson's *r*), indicating how they were calculated

Our web collection on statistics for biologists contains articles on many of the points above.

Software and code

Policy information about availability of computer code

Data collection	Mass spectrometry data was acquired using the Orbitrap Astral MS, using Thermo Tune software (version: 0.4 or higher)
Data analysis	Proteomics data was analysed using Spectronaut (v17 and v18) as well as DIA-NN (v1.8.1). Raw data was also processed using MaxQuant (v1.6.7.0 and v1.6.14.0).Spectronaut output was reformatted using the Perseus plugin peptide collapse (Hogrebe et al 2018) to create a MaxQuant-like site-table. Protein grouping was performed using default protein inference workflow with IDpicker as inference algorithm.Raw files from DIA and DDA comparison experiments were analyzed in DIA-NN 1.8.1. Plots from figures 1b-d were based on MS1 feature detection output retrieved by MaxQuant (v1.6.7.0). Total Ion Current intensity for figure 3c were obtained from MaxQuant (v1.6.14.0). Each fractionation scheme was search independently except for searches performed in triplicates. Fractionation quantification was performed using MaxLFQ algorithm embedded in iq R package (Pham et. al 2020). To determine the percent of residues in each identified protein sequence (sequence coverage), the program Protein Coverage Summarizer (PNNL-Comp-Mass-Spec) was used. Enrichment analisys was performed with the package ClusterProfiler (Wu et. al , 2021) and using the org.Sc.sgd.db and org.Hs.eg.db. All data analysis was performed using R v 4.2.2 and R studio v 2022.12.0 Build 353

For manuscripts utilizing custom algorithms or software that are central to the research but not yet described in published literature, software must be made available to editors and reviewers. We strongly encourage code deposition in a community repository (e.g. GitHub). See the Nature Portfolio guidelines for submitting code & software for further information.

Data

Policy information about availability of data

All manuscripts must include a data availability statement. This statement should provide the following information, where applicable:

- Accession codes, unique identifiers, or web links for publicly available datasets
- A description of any restrictions on data availability
- For clinical datasets or third party data, please ensure that the statement adheres to our policy

The mass spectrometry proteomics data have been deposited to the ProteomeXchange Consortium via the PRIDE partner repository with the dataset identifiers: (i)DDA vs DIA comparison: PXD046453 (ii) fractionation strategies: PXD046372, (iii) three species mix: PXD046444, (iv) yeast KO collection: PXD046386, (v) clinical samples(MSA vs Ctrl) : PXD046417,(vi) single cell data: PXD046357,(vii) single shot dilution series: PXD046283 and (viii) window optimization: PXD046285. Supplementary table 5 contains an overview of the experiments.. Uniprot reference human proteome used in Spectronaut searches was 2022 release (20,598 entries).

Human research participants

Policy information about studies involving human research participants and Sex and Gender in Research.

Reporting on sex and gender	The number of samples from each sex was balanced good as possible between the MSA group (M = 21, F = 24) and the Control Group (M = 13, F = 16) taking into account the limited number of samples available. Sex as a factor was not considered in evaluation of the results of the MSA study.
Population characteristics	Race, ethnicity of other social variables were not taken into account in the study.The human brains analyzed in the current study were donated to the Brain Bank at Bispebjerg-Frederiksberg Hospital (Copenhagen University Hospital, Denmark) or to the MRC London Neurodegenerative Diseases Brain Bank (King's College London, United Kingdom). All donated brains were neuropathologically examined to verify the diagnosis. In total, brains from 29 individuals showing no signs of neuropathological disease and 45 MSA patients were included in the analysis
Recruitment	The human brains utilized in the current study were donated to the Brain Bank (n = 38) at Bispebjerg-Frederiksberg Hospital (Copenhagen University Hospital, Denmark) or to the MRC London Neurodegenerative Diseases Brain Bank (n= 36) at King's College London, United Kingdom.
Ethics oversight	Collection and analysis of the samples was conducted in accordance with the World Medical Association Declaration of Helsinkiand approved by the regional ethical committee of the Capitol Region (Denmark), j.nr. H-16025210 and H-15016232 and the Danish data protection agency (j.nr. P-2020-937).

Note that full information on the approval of the study protocol must also be provided in the manuscript.

Field-specific reporting

Please select the one below that is the best fit for your research. If you are not sure, read the appropriate sections before making your selection.

☒ Life sciences ☐ Behavioural & social sciences ☐ Ecological, evolutionary & environmental sciences

For a reference copy of the document with all sections, see nature.com/documents/nr-reporting-summary-flat.pdf

Life sciences study design

All studies must disclose on these points even when the disclosure is negative.

Sample size	To assess technical variability of the mass spectrometry measurements each experiment was performed using at least three injection replicates. In the clinical profiling experiment, we used 74 biological independent samples.
Data exclusions	No data was excluded from the analysis except for 11 identified outliers in the MSA cohort depicted in Supplementary Figure 9 recapitulating the fundings of the original data set (Rydbirk, Østergaard Folke et al., 2022)
Replication	All experiments were performed using replicates as stated in the sample size section. All the replication attempts were succcesful
Randomization	No randomization was performed for technical benchmark, since the performance of the instrument was tested and the technical replicates were used to asses precision and accuracy rather than comparing treatment effects. Clinical samples were randomized before injection.
Blinding	Researchers were not blinded to perform the experiments reported in this work.<br>This was required as to keep a strict overview of mass spectrometry method selection.<br>When validating the performance of an instrument or equipment, technical replicates may be used to assess precision and accuracy.<br>Randomization may not be relevant in this case as the focus is on evaluating the instrument's capability rather than comparing treatment

effects

Reporting for specific materials, systems and methods

We require information from authors about some types of materials, experimental systems and methods used in many studies. Here, indicate whether each material, system or method listed is relevant to your study. If you are not sure if a list item applies to your research, read the appropriate section before selecting a response.

Materials & experimental systems

n/a	Involved in the study
☒	☐ Antibodies
☐	☒ Eukaryotic cell lines
☒	☐ Palaeontology and archaeology
☒	☐ Animals and other organisms
☒	☐ Clinical data
☒	☐ Dual use research of concern

Methods

n/a	Involved in the study
☒	☐ ChIP-seq
☒	☐ Flow cytometry
☒	☐ MRI-based neuroimaging

Eukaryotic cell lines

Policy information about cell lines and Sex and Gender in Research

Cell line source(s)	ATCC (HeLa, HEK293)
Authentication	The cell lines used were not autenticated
Mycoplasma contamination	The cell lines were tested for mycoplasma contamination
Commonly misidentified lines (See ICLAC register)	N/A
